# Depletion of IQ motif-containing GTPase activating protein 2 (IQGAP2) reduces hepatic glycogen and impairs insulin signaling

**DOI:** 10.1016/j.jbc.2023.105322

**Published:** 2023-10-05

**Authors:** Anushna Sen, Sara Youssef, Karen Wendt, Sayeepriyadarshini Anakk

**Affiliations:** 1 Department of Molecular and Integrative Physiology, University of Illinois at Urbana-Champaign, Urbana, Illinois, USA; 2 Division of Nutritional Sciences, University of Illinois at Urbana-Champaign, Urbana, Illinois, USA; 3 Cancer Center at Illinois, University of Illinois at Urbana-Champaign, Urbana, Illinois, USA

**Keywords:** scaffold protein, liver, carbohydrate, glycogen synthase (GYS2), protein kinase B (PKB/AKT), glycogen synthase kinase 3 (GSK-3), insulin signaling

## Abstract

The liver is critical in maintaining metabolic homeostasis, regulating both anabolic and catabolic processes. Scaffold protein IQ motif-containing GTPase activating protein 2 (IQGAP2) is highly expressed in the liver and implicated in fatty acid uptake. However, its role in coordinating either fed or fasted responses is not well understood. Here we report that IQGAP2 is widely expressed in the liver that is pronounced in the pericentral region. Although control and IQGAP2 knockout mouse model showed comparable hepatic gene expression in the fasted state, we found significant defects in fed state responses. Glycogen levels were reduced in the periportal region when IQGAP2 was deleted. Consistently, we observed a decrease in phosphorylated glycogen synthase kinase 3α and total glycogen synthase protein in the fed IQGAP2 knockout mice which suggest inadequate glycogen synthesis. Moreover, immunoprecipitation of IQGAP2 revealed its interaction with GSK3 and GYS. Furthermore, our study demonstrated that knocking down IQGAP2 *in vitro* significantly decreased the phosphorylation of AKT and forkhead box O3 proteins downstream of insulin signaling. These findings suggest that IQGAP2 contributes to liver fed state metabolism by interacting with glycogen synthesis regulators and affecting the phosphorylation of insulin pathway components. Our results suggest that IQGAP2 plays a role in regulating fed state metabolism.

The liver tightly regulates lipid and glucose metabolism to maintain energy homeostasis (1, 2) and is programmed to adapt to nutrient fluctuations. During the fed state, insulin signaling regulates glucose uptake, glycogenesis, and *de novo* lipogenesis in the liver. Excess carbohydrates are stored as glycogen with glycogen synthase (GYS2) and its regulator glycogen synthase kinase 3 (GSK3) governing this process (1, 3). Also, under nutrient excess, sterol response element binding protein 1c (SREBP-1c*)* transcriptionally regulates fatty acid synthase (*Fasn*) to promote *de novo* lipogenesis (3, 4). Under fed conditions, fatty acids are stored as triglycerides, with diacylglycerol acyltransferases (DGAT2) catalyzing the final step of triglyceride synthesis (5). Upon fasting, glycogen stores are depleted, and fatty acid oxidation (6, 7, 8) is switched on.

Over the past decades, scaffold protein-mediated metabolic regulation has gained relevance since these proteins can facilitate the integration of multiple signaling pathways (9). The IQ motif-containing GTPase activating protein (IQGAP) scaffold protein family consists of three highly homologous members (IQGAP1, IQGAP2, and IQGAP3), with IQGAP1 being the most studied isoform (10, 11, 12, 13, 14, 15, 16, 17). While IQGAP1 is ubiquitously expressed, IQGAP2 is mainly expressed in the liver and platelets (18). We recently showed that IQGAP1 was crucial for regulating ketogenesis under fasting conditions (16). Here, we investigate IQGAP2’s role in hepatic metabolism (fed *versus* fasted state) since SNP variants of the human IQGAP2 gene are associated with diabetes mellitus (19), and the IQGAP2 knockout (*Iqgap2*^*−/−*^) mouse model showed perturbed metabolic homeostasis with altered hepatic fatty acid uptake and lipid processing (20, 21).

## Results

### IQGAP2 is expressed throughout the liver

**Figure 1:**
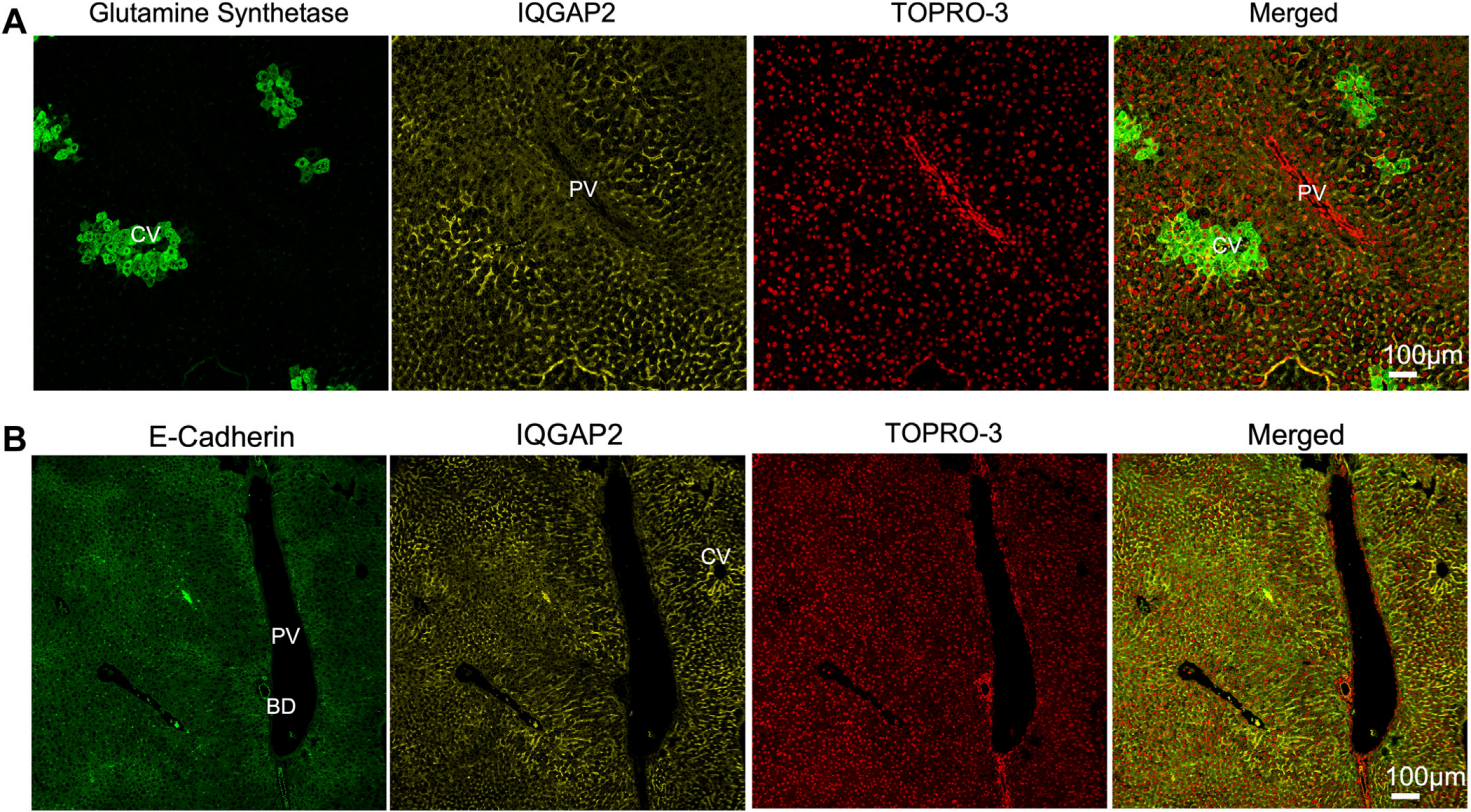
**IQGAP2 localization within the liver.** IQGAP2 distribution within wildtype mice liver lobules was examined with immunohistochemistry. *A* and *B*, pericentrally expressed (*A*) glutamine synthetase and periportally expressed E-cadherin (*B*) were used to identify liver zonation. IQGAP2 was expressed in the hepatocytes and increased around the central vein while absent from the bile duct cells. BD, bile duct; CV, central vein; PV, portal vein. TOPRO-3, nuclear stain. N = 3 to 4 mice per group. 100 μm. IQGAP2, IQ motif-containing GTPase activating protein 2.

Metabolic functions in the liver are zonally distributed. We examined the expression and localization of murine IQGAP2 using immunohistochemistry. IQGAP2 expression was noted in the entire liver and sinusoidal areas except for the bile ducts. Then, we compared the spatial distribution of IQGAP2 with zonally expressed proteins, E-cadherin (periportal marker) and glutamine synthetase (pericentral marker) in the liver (22). Double-immunofluorescence staining of wildtype (WT) livers showed more robust co-expression of IQGAP2 with the pericentral marker glutamine synthetase (Fig. 1*A*) rather than periportal E-cadherin (Fig. 1*B*). Based on the IQGAP2 expression pattern, we then examined if IQGAP2 is needed to coordinate the fed and fasted state response in the liver.

### A decrease in the liver-to-body weight ratio was noted only in *Iqgap2*^*−/−*^ female mice

Compared to WT controls, male *Iqgap2*^*−/−*^ mice did not reveal any change in their liver or adipose weight to body weight ratios, as shown in Table 1. However, *Iqgap2*^*−/−*^ females showed decreased liver-to-body weight ratio both in fed and fasted states. First, we examined if the loss of the *Iqgap2* gene was compensated by overexpression of *Iqgap1* and/or *Iqgap3* transcript and found no evidence for it in *Iqgap2*^*−/−*^ mice (Fig. S1, *A*–*F*). Next, we characterized the liver histology and found no obvious difference between the WT and *Iqgap2*^*−/−*^ mice (Fig. S1*G*) or between the fed and fasted states, indicating the loss of IQGAP2 does not alter overall liver architecture.

**Table 1:** Liver and white adipose tissue (WAT) organ weights with respect to the body weights (BW) of fed and 24-h fasted wildtype and *Iqgap2*^*−/−*^ mice (males and females, 16–20 weeks old)

Gross morphology	Wildtype fed	*Iqgap2*^−/−^ fed	Wildtype fasted	*Iqgap2*^−/−^ fasted
	N = 7–9	N = 6–7	N = 9	N = 5–9
Males				
Body weight	27.62 ± 2.39	29.48 ± 3.47	23.76 ± 3.97	25.51 ± 2.84
Liver: BW %	3.97 ± 0.44	3.47 ± 0.51	3.18 ± 0.75^a^	2.84 ± 0.51^a^
WAT: BW %	2.76 ± 0.9	2.7 ± 0.75	2.37 ± 0.52	2.68 ± 1.17
Females				
Body weight	22.77 ± 2.12	22.81 ± 1.98	19.90 ± 2.43	24.44 ± 4.15^b^
Liver: BW %	3.94 ± 0.8	3.17 ± 0.25^b^	3.4 ± 0.39	2.91 ± 0.16^b^
WAT: BW %	2.94 ± 0.96	2.15 ± 0.97	1.93 ± 0.83	2.39 ± 1.13

Statistics were calculated using unpaired *t* test.

a *p* < 0.05 *versus* Fed control.

b *p* < 0.05 *versus* WT control.

### Loss of IQGAP2 resulted in reduced expression of genes involved in *de novo* lipogenesis

**Figure 2:**
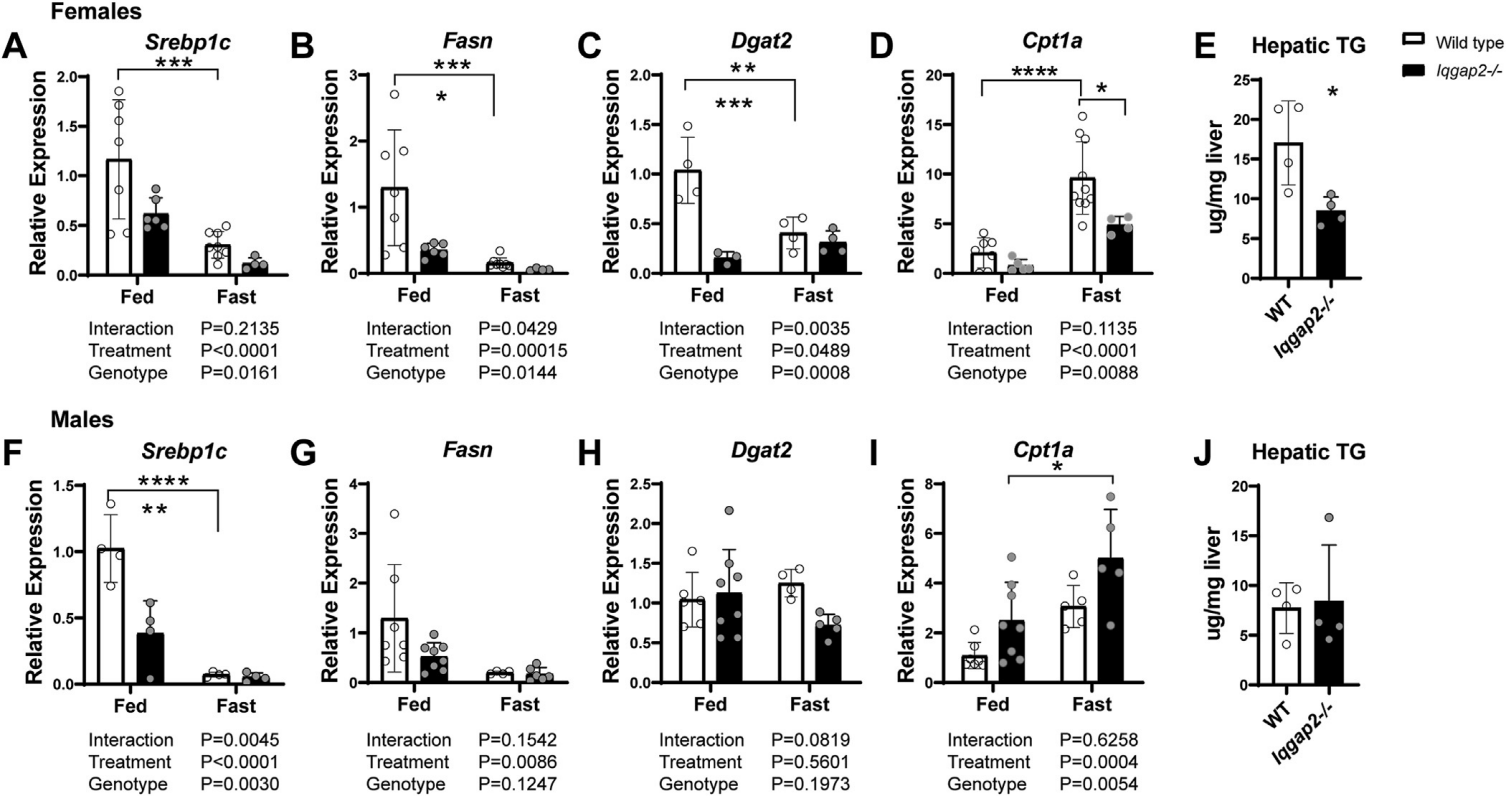
**Depletion of *Iqgap2* blunts hepatic lipid metabolism genes in female mice.***Iqgap2*^*−/−*^ and wildtype mice (male and female, 16–20 weeks old, n = 5–9) were fed regular chow ad libitum or fasted for 24 h. Liver tissues were analyzed with qRT-PCR to quantify the mRNA of key genes involved in lipid metabolism. *A* and *F*, lipogenic transcription factor *Srebp1c* was downregulated in both female and male *Iqgap2*^*−/−*^ mice. *B*–*C*, *G* –*H*, however, exhibit sex differences, (*B*) *Fasn* and (*C*) *Dgat2* were reduced in female but not in *Iqgap2*^*−/−*^ male mice (*G* and *H*). Hepatic triglyceride levels were decreased in *Iqgap2*^*−/−*^ female (*E*) but not male mice (*J*). *D*, fasting-mediated increases in the expression of *Cpt1a gene,* crucial in fatty acid oxidation, was diminished in *f*emale *Iqgap2*^*−/−*^ mice. In contrast, male mice showed significant upregulation *of Cpt1a* upon fasting (I). Statistics were calculated using two-way ANOVA with Bonferroni post hoc analysis. ∗*p* < 0.05, ∗∗*p* < 0.01, ∗∗∗*p* < 0.001, ∗∗∗∗*p* < 0.0001. *Fasn,* fatty acid synthase; 3; IQGAP2, IQ motif-containing GTPase activating protein 2; *Iqgap2*^*−/−*^, IQGAP2 knockout; *Srebp1c,* sterol response element binding protein 1c.

Lipid metabolism is altered between the fed and fasted states (3); therefore, we explored if IQGAP2 loss influenced this process. Both sexes of *Iqgap2*^*−/−*^ mice displayed reduced mRNA expression of *Srebp1c* (Fig. 2, *A* and *F*), with females showing a significant reduction of *Fasn* and *Dgat2,* key downstream regulators of lipogenesis, compared to WT livers (Fig. 2, *B* and *C*). Consistently, *Iqgap2*^*−/−*^ females displayed significantly lower fed state hepatic triglyceride content (Fig. 2*E*), but this was not observed in *Iqgap2*^*−/−*^ male mice (Fig. 2, *G*, *H*, and *J*). Also, induction of *Cpt1a* gene expression during fasting was blunted only in *Iqgap2*^*−/−*^ female mice (Fig. 2, *D* and *I*), indicating a sex-specific effect. To test if IQGAP2 expression *per se* is sexually dimorphic, which could explain this result, we tested the estrogen signaling axis. We examined ERα-expressing HepG2 cells treated with either estradiol or ethinyl estradiol, as well as sham and ovariectomized mice for IQGAP2 protein levels (Fig. S2, *C*–*F*). Although we found a slight reduction in IQGAP2 levels after ovariectomy, it was not statistically significant (Fig. S2, *E* and *F*). We were also unable to increase IQGAP2 levels with estradiol agonism. Additionally, hepatic IQGAP2 protein levels in both sexes of WT mice in fed and fasted states were comparable (Fig. S2, *A* and *B*). These findings indicate that the sex-specific response noted in *Iqgap2*^*−/−*^ mice is not due to the regulation of IQGAP2 expression by the estrogen axis.

### *Iqgap2*^*−/−*^ mice exhibit intact mitochondrial gene expression pattern

**Figure 3:**
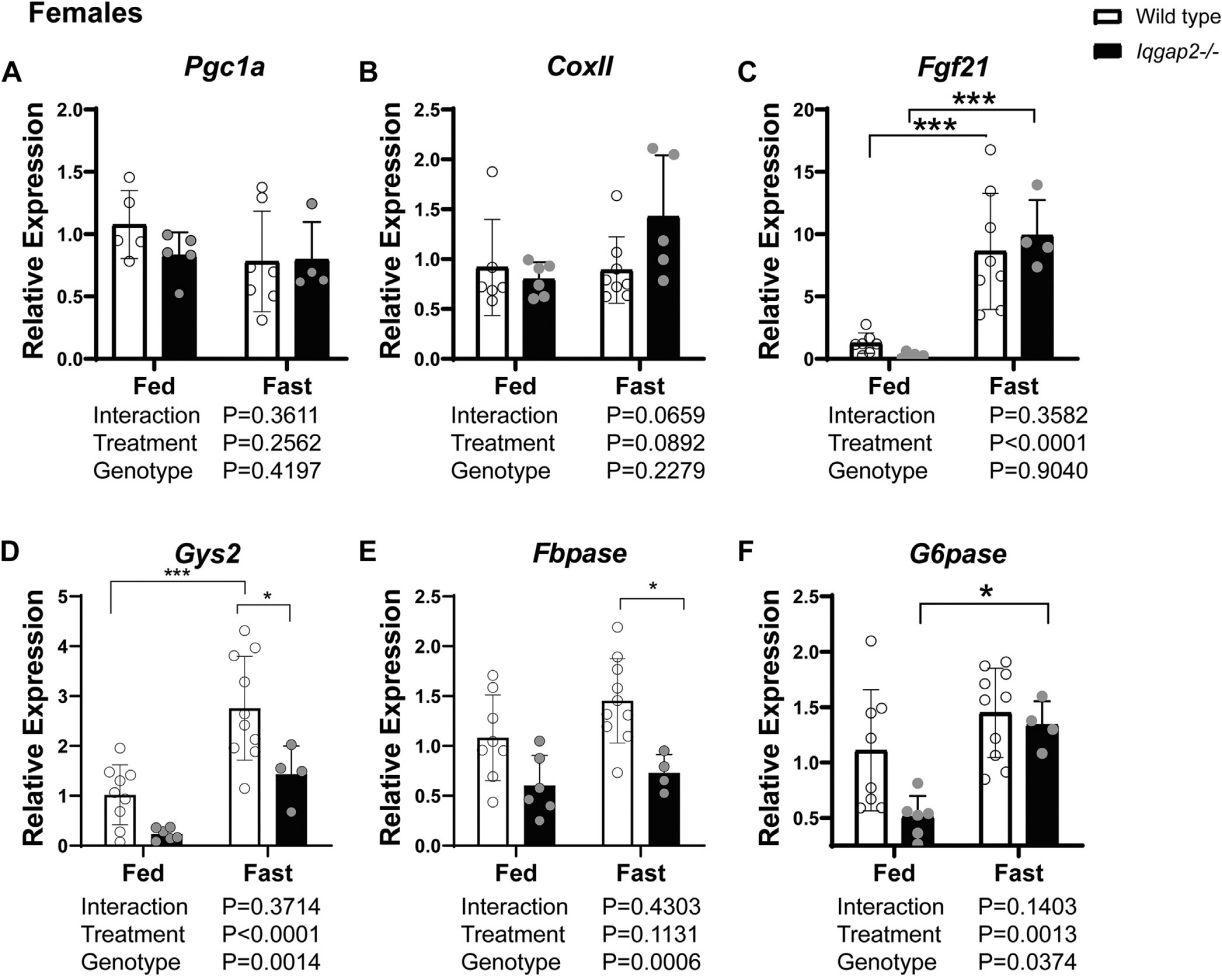
**Expression of genes involved in carbohydrate metabolism is altered in female *Iqgap2***^***−/−***^**mice.** Liver tissues from *Iqgap2*^*−/−*^ and wildtype mice were examined to quantify the expression of genes involved in mitochondrial function, fasting response, glycogen synthesis, and gluconeogenesis. *A* and *B*, transcript levels of the master regulator of mitochondrial biogenesis (*A*) *Pgc1-α* and mitochondrial complex II (*B*) were similar between the groups. *C*, fasting response *Fgf21* gene expression was comparable in the fed state, and the fasting-mediated increase in *Fgf21* was unaltered in *Iqgap2*^*−/−*^ mice. *D*, glycogen synthase (*Gys2*) gene expression was decreased in *Iqgap2*^*−/−*^ mice. *E*, female *Iqgap2*^*−/−*^ mice also showed blunted gluconeogenic response upon fasting with decreased expression of *Fbpase. F*, *G6pase* expression was lower in the fed state, but the fasted response was comparable to wildtype controls. Statistics were calculated using two-way ANOVA with Bonferroni post hoc analysis. ∗*p* < 0.05, ∗∗∗*p* < 0.001. N = 5 to 9 mice per group. IQGAP2, IQ motif-containing GTPase activating protein 2; *Iqgap2*^*−/−*^, IQGAP2 knockout.

We next examined the expression of crucial mitochondrial genes such as the peroxisome proliferator-activated receptor-γ coactivator 1-α (*Pgc1a*) (23, 24) and the mitochondrial cytochrome c oxidase subunit II and mitochondrial cytochrome c oxidase subunit III (Fig. S3*C*). There was no difference in their expression pattern between WT and *Iqgap2*^*−/−*^ mice (Fig. 3, *A* and *B*). Since fasting responses can be regulated by fibroblast growth factor 21 (FGF21) (25, 26, 27, 28), we tested and observed an appropriate fasting-mediated *Fgf21* induction (Fig. 3*C*) along with comparable levels of its cognizant receptor *Fgfr1*and adaptor β-Klotho (*Klb*) (Fig. S3, *A* and *B*) between WT and *Iqgap2*^*−/−*^ mice. These results suggest that the fasting response and mitochondrial gene expression are maintained in the absence of IQGAP2 expression.

### Impaired expression of glucose metabolism genes is noted in *Iqgap2*^*−/−*^ livers

To evaluate whether loss of IQGAP2 affects carbohydrate metabolism, we analyzed genes regulating glycogenesis (*Gys2*), gluconeogenesis (glucose 6-phosphatase, *G6pase,* and fructose 1,6-bisphosphatase, *Fbpase*), and glycolysis (phosphofructokinase, *Pfkl*, and glucose transporters 1 and 2, *Glut1* and *Glut2,* respectively) (29, 30, 31). *Iqgap2*^*−/−*^ female mice displayed compromised gene expression in these pathways, primarily in the fed state. *Iqgap2*^*−/−*^ female livers displayed a striking five-fold decrease in *Gys2* mRNA levels (Fig. 3*D*), while *Iqgap2*^*−/−*^ male mice displayed a modest increase in *Gys2* compared to their WT controls (Fig. S4*A*). Further, we observed a decreasing expression pattern of gluconeogenic genes, with 2-fold lower *Fbpase* and *G6pase* in *Iqgap2*^*−/−*^ females (Fig. 3, *E* and *F*) but not in the male mice (Fig. S4, *B* and *C*). However, glycolysis and glucose transport genes remained unaltered between *Iqgap2*^*−/−*^ and WT mice (Fig. S3, *D* and *F*). As *de novo* lipogenic and glycogenic gene expression was distinctly altered in female *Iqgap2*^*−/−*^ mice, we performed further analysis using the female mice.

### Depletion of hepatic glycogen is observed in *Iqgap2*^*−/−*^ female livers

**Figure 4:**
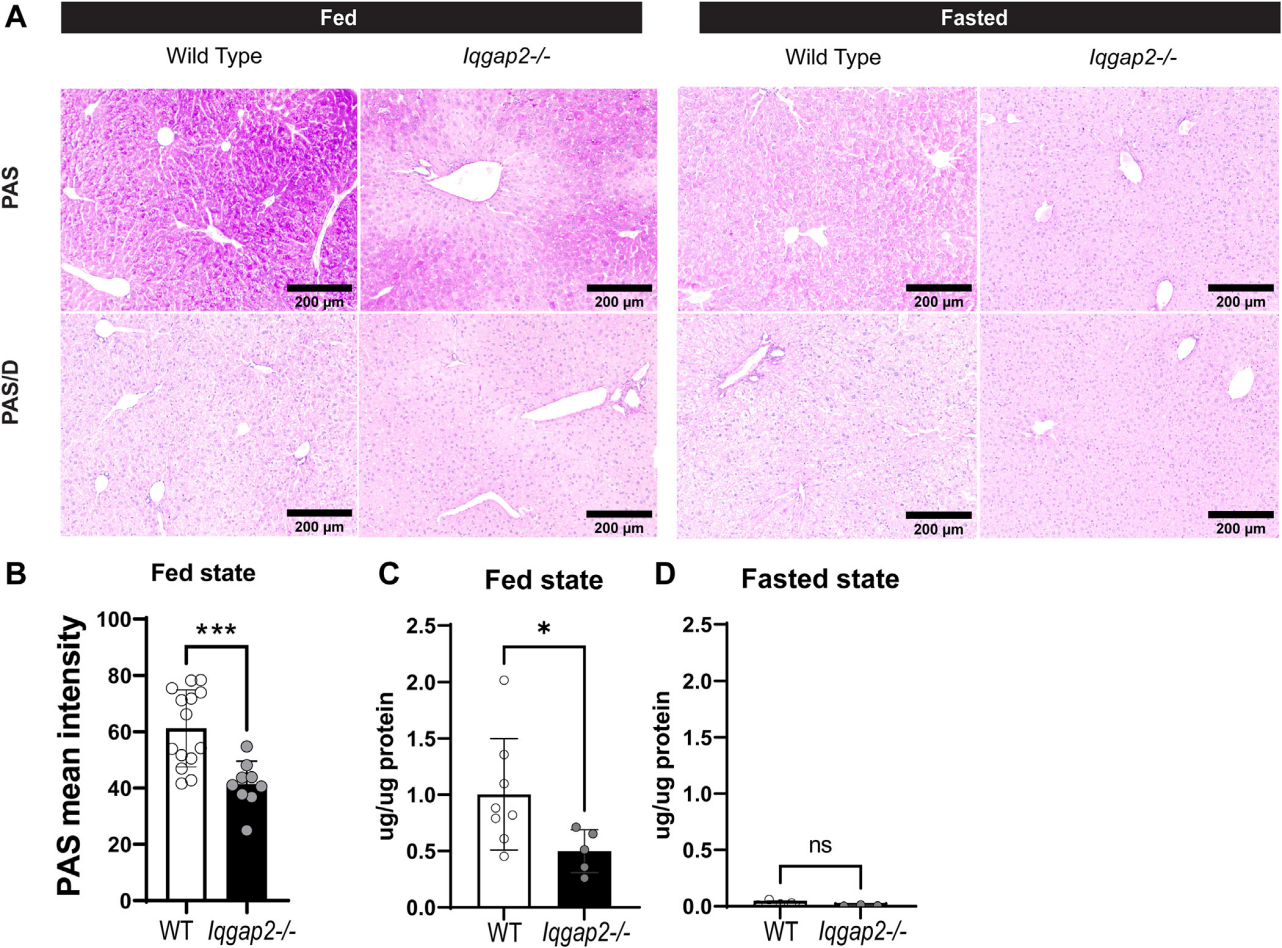
**Lower hepatic glycogen level is seen in *Iqgap2***^***−/−***^**female livers.***Iqgap2*^*−/−*^ and WT mice were fed regular chow ad libitum. Liver tissue sections were analyzed for glycogen levels by periodic acid-Schiff stain (PAS). *A*, histological examination demonstrates the uniform distribution of hepatic glycogen in the fed state of (*A*) WT mice, whereas periportal glycogen depletion is apparent in *Iqgap2*^*−/−*^ mice. Each panel represents an individual mouse. Diastase treatment digests glycogen, so PAS/D+PAS is used to examine glycogen levels. *B* and *C*, liver glycogen was quantified using PAS intensity (*B*) and biochemical assay (*C* and *D*). Statistics were calculated using Welch’s *t* test analysis and two-way ANOVA with Bonferroni post hoc analysis. ∗*p* < 0.05, ∗∗∗*p* < 0.001. N = 3 to 5 mice per group. 200 μm. IQGAP2, IQ motif-containing GTPase activating protein 2; *Iqgap2*^*−/−*^, IQGAP2 knockout.

We then validated the observed defects in the gene expression profile of glycogen synthesis in IQGAP2-deficient mice by periodic acid-Schiff (PAS)–based staining of glycogen levels in WT and *Iqgap2*^*−/−*^ livers (Fig. 4). Hepatic glycogen was uniformly distributed in WT fed mice; however, glycogen staining was significantly depleted around the periportal regions in *Iqgap2*^*−/−*^ mice in the fed state. We performed a PAS stain in the presence and absence of diastase (Fig. 4, *A* and *B*) to differentiate between glycogen and glycoproteins as diastase digests glycogen. Upon diastase treatment, we did not see any difference in PAS levels between WT and *Iqgap2^−/−^* livers, validating the loss of glycogen around the periportal areas. We quantified glycogen levels from both sexes of mice using a biochemical assay and found a 38% to 48% decrease in *Iqgap2*^*−/−*^ mice in the fed state (Fig. 4*C*), which was significant only in female but not male mice. Although periportal glycogen depletion is seen in *Iqgap2*^−/−^ males (Fig. S5*A*), it is visibly exaggerated in *Iqgap2*^*−/−*^ female livers in the fed state (Fig. 4*A*). It is important to note that fasting-induced glycogen depletion was similar between WT and *Iqgap2*^*−/−*^ mice, suggesting that mobilization of glycogen remained intact even in the absence of IQGAP2 (Figs. 4*D* and S5*C*).

### IQGAP2 modulates GYS2 levels and GSK3 phosphorylation

**Figure 5:**
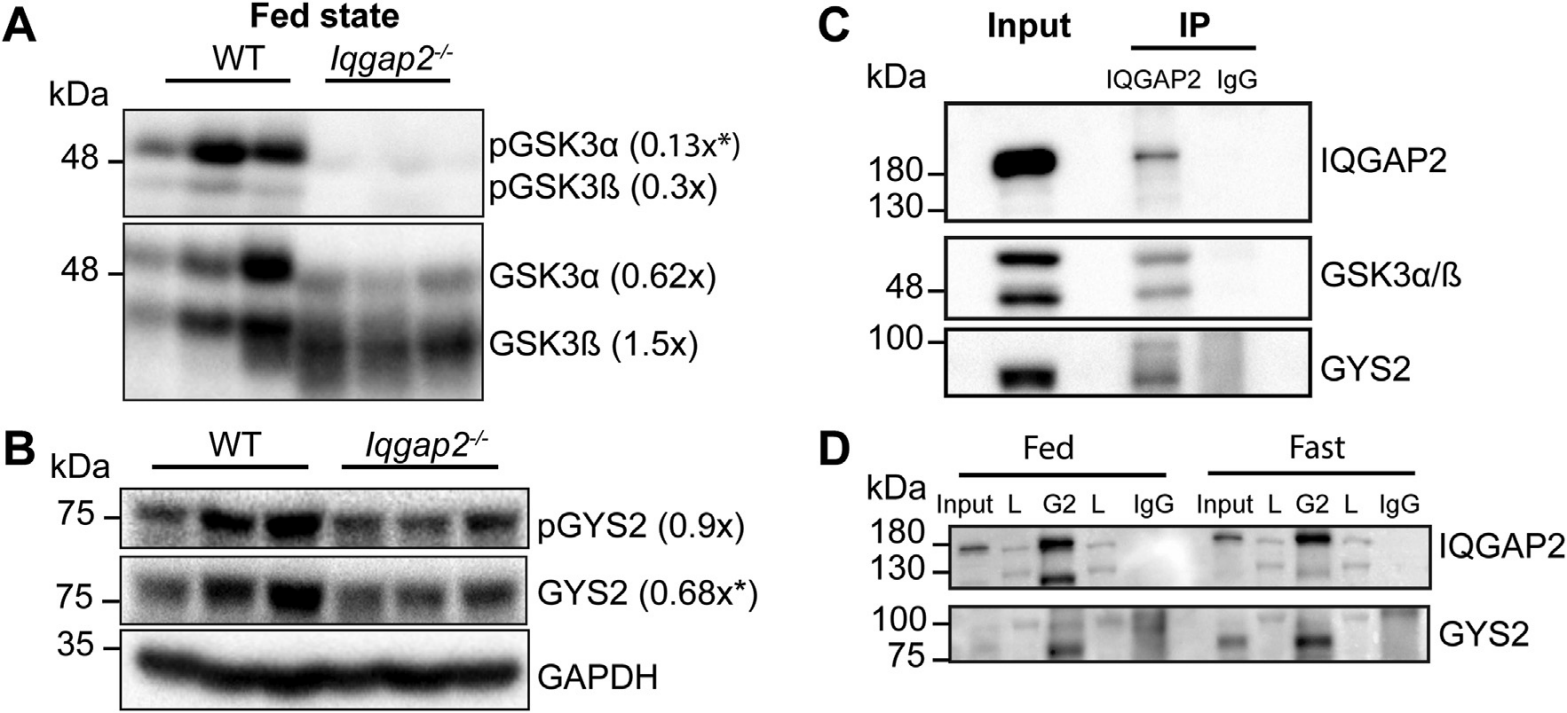
**Iqgap2 depletion results in reduced inactivation of GSK3 signaling and decreased GYS2 expression.** Liver tissues from female *Iqgap2*^*−/−*^ and WT mice were examined by Western blot analysis. *A*, hepatic total GSK3α/β and phosphorylated GSK3α/β (ser21/9) protein levels were measured by western blots. Densitometric analysis of inactive GSK3α (ratio of pGSK to GSK) indicated increased GSK3α activity in *Iqgap2*^*−/−*^ mice, whereas inactive GSK3β activity was not significantly decreased. To validate this increased GSK3α activity, the phosphorylation of its downstream target GYS2 was examined in *Iqgap2*^*−/−*^ mice. *B*, GYS2 phosphorylation was comparable between *Iqgap2*^*−/−*^ and WT mice. However, total GYS2 protein expression, was decreased in *Iqgap2*^*−/−*^ mice. *C*, to test where IQGAP2 directly interacts with GSK3 and GYS2, IQGAP2 complexes in HEK293T cell lysate were probed for co-immunoprecipitation with GSK3α/β and GYS2. HEK293 cell lysates were incubated with IQGAP2 or IgG antibody (negative control). *D*, IQGAP2 was also immunoprecipitated from fed and fasted livers and its interaction with GYS2 was tested. G2, IQGAP2 pull down; L, Ladder. 1% of the cell lysate was loaded onto the gel as input. IP- immunoprecipitation. Glyceraldehyde-3- phosphate dehydrogenase (GAPDH) served as a loading control for immunoblot analysis. Statistics were calculated using Student’s *t* test analysis. ∗*p* < 0.05. N = 3 to 4 mice per group. GSK3, glycogen synthase kinase 3; GYS2, glycogen synthase; IQGAP2, IQ motif-containing GTPase activating protein 2; *Iqgap2*^*−/−*^, IQGAP2 knockout.

Because IQGAP2 deletion reduced glycogen levels, we analyzed the protein expression of both GYS2 and GSK3. GYS2 levels were significantly reduced in the *Iqgap2*^*−/−*^ fed livers compared to the WT (Fig. 5*B*), indicating that IQGAP2 plays an important role in glycogenesis. Total levels of GSK3α, which is responsible for regulating GYS2 activity (3), were also lower (Fig. 5*A*) but did not reach statistical significance. However, when we tested the activity of GSK3α/β by measuring their phosphorylated forms, we found that both p-GSK3α and β levels were reduced (Fig. 5*A*). This decrease in GSK3 phosphorylation signifies its increased activation in *Iqgap2*^*−/−*^ livers, which in turn correlates with the reduced glycogen accumulation. We then tested if IQGAP2 could bind with GSK3 and GYS2. Immunoprecipitation of IQGAP2 complexes from HEK293T cells confirmed physical interaction between IQGAP2, GSK3, and GYS2 (Fig. 5*C*). We also found IQGAP2 interaction with GYS2 in WT mouse livers in fed and fasted states (Fig. 5*D*). These results indicate that misregulation of GSK3 and GYS2 may explain the depletion of hepatic glycogen observed even in the fed state of the *Iqgap2*^*−/−*^ female mice.

### IQGAP2 knockdown results in defective insulin signaling *in vitro*

**Figure 6:**
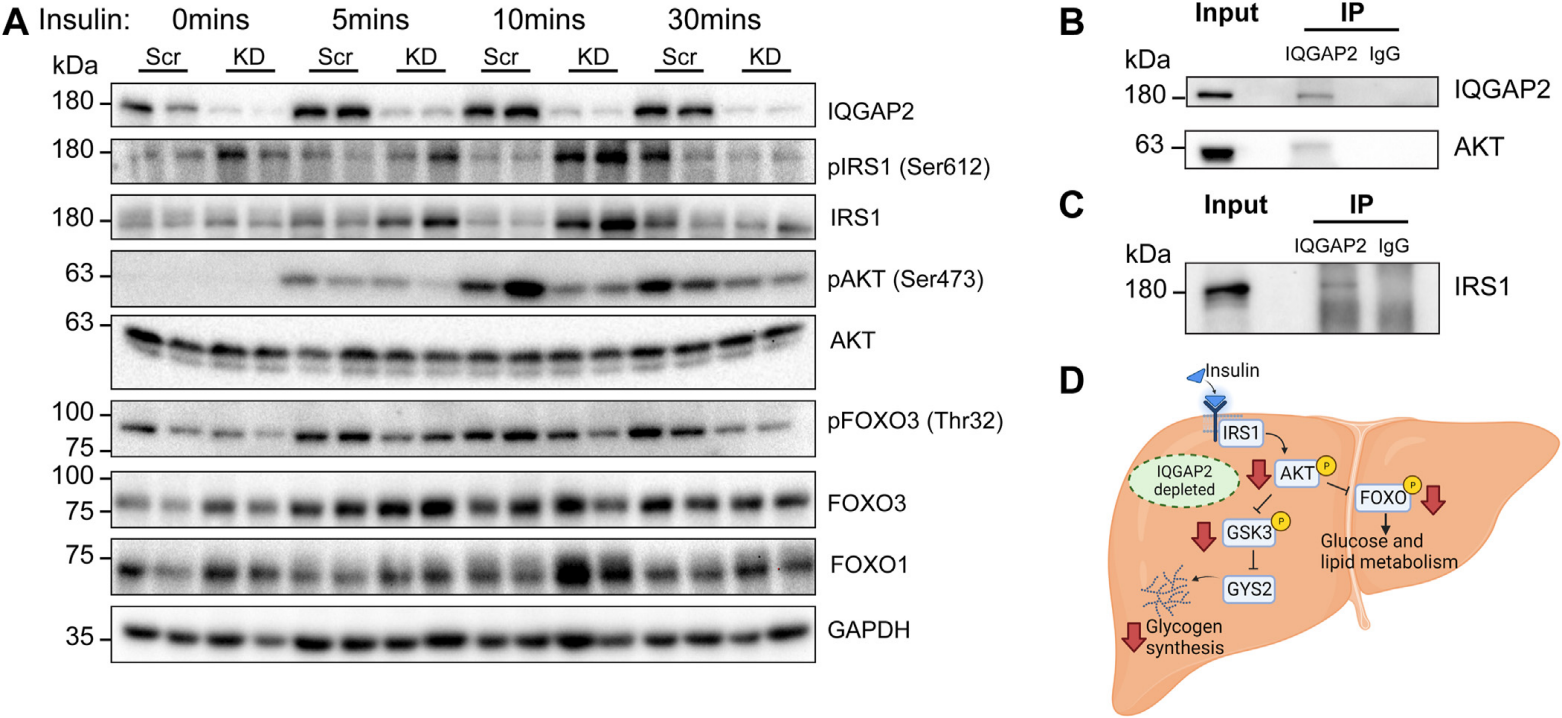
**IQGAP2 knockdown impairs insulin downstream signaling.** To examine whether IQGAP2 blunts insulin response, HepG2 liver cells were transfected with scrambled or IQGAP2 shRNA before being treated with insulin (100 nM for 5, 10, or 30 min). *A*, changes in the insulin signaling pathway were examined by Western blot analysis. IQGAP2 expression decreased by 90% after knockdown in HepG2 cells. IQGAP2 depletion increased IRS1 expression though phosphorylation of IRS1 was comparable between control and IQGAP2 knockdown samples. Insulin stimulation of AKT phosphorylation was decreased in IQGAP2-depleted samples. Phosphorylation of downstream target FOXO3 was also significantly reduced in IQGAP2 knockdown samples. *B* and *C*, HEK293 cell samples were immunoprecipitated with anti-IQGAP2 or rabbit IgG antibodies, and immunoblots were probed for AKT and IRS1. *D*, schematic depicting how IQGAP2 depletion affects liver metabolism. Scr, scramble shRNA; KD, knockdown with IQGAP2 shRNA; IP, immunoprecipitation. Schematic created with BioRender.com. FOXO, forkhead box O; GSK3, glycogen synthase kinase 3; GYS2, glycogen synthase; IQGAP2, IQ motif-containing GTPase activating protein 2; *Iqgap2*^*−/−*^, IQGAP2 knockout; IRS, insulin receptor substrate.

Based on the results, it suggested that loss of IQGAP2 may affect the fed state metabolic signals. Therefore, we examined insulin downstream signals, including total and phosphorylated IRS1, AKT, and their downstream targets, forkhead box O1 (FOXO1) and FOXO3a (Fig. 6). Insulin receptor substrate (IRS) proteins recruit effector proteins that activate AKT upon insulin stimulation. Upon IQGAP2-depletion, IRS1 protein levels significantly increased within 5 min of insulin stimulation and remained elevated for 10 min (Fig. 6*A*). More importantly, IQGAP2 knockdown significantly blunted insulin-mediated AKT activation by ∼70% (Figs. 6*A* and S6*E*). This was pronounced at the 10 min post insulin treatment. Immunoprecipitation of IQGAP2 revealed physical interaction between IQGAP2, IRS1, and AKT in HEK293T cells (Fig. 6, *B* and *C*). Finally, to confirm this AKT signaling defect, we examined downstream targets, FOXO1 and FOXO3a. During the fed state, AKT is responsible for inhibitory phosphorylation of FOXO proteins. Subsequently, we found increased total FOXO1 and significantly reduced inhibitory phosphorylation of FOXO3a (Thr32) upon IQGAP2 knockdown (Figs. 6*A* and S6*H*), which is consistent with defective insulin and AKT signaling (Fig. 6*D*).

## Discussion

Disruption of liver metabolism is linked to insulin resistance and diabetes. Thus, understanding molecular regulators of liver metabolism is essential. Over the past few years, the IQGAP family has been shown to affect liver metabolism (16, 20, 21, 32). Here, we investigated whether IQGAP2 coordinates metabolic signaling pathways within the liver. We uncovered a role for IQGAP2 in hepatic glycogen synthesis. Using IQGAP2-knocked down cells, we demonstrate IQGAP2 is required for normal insulin response and that IQGAP2 may scaffold various components of the insulin signaling pathway.

The liver’s metabolic functions are not uniformly distributed but occur nonhomogenously across ‘zones’ along the portocentral axis in the liver. IQGAP2 was expressed in both zone 1 and zone 3 hepatocytes, with elevated pericentral expression. This finding is consistent with the scRNA-seq data, wherein the *Iqgap2* transcript was found to be a central zone marker (33). IQGAP2 zonal expression corroborates its role in metabolic processes, such as lipogenesis, triglyceride synthesis, and glycogen synthesis from glucose. Glycogen depletion in *Iqgap2*^*−/−*^ livers was prominent in periportal hepatocytes, which synthesize glycogen indirectly from gluconeogenic precursors, indicative of a defect in this pathway when IQGAP2 is lost (34). Hence, we postulate that IQGAP2 predominantly regulates anabolic processes during the fed state liver.

The unexpected decrease of ∼ 38% hepatic glycogen in *Iqgap2*^*−/−*^ female livers correlates well with the ∼30% GYS2 expression reduction. Notably, liver GYS2 knockout mice display ∼95% reduction of hepatic glycogen (35), highlighting its relevance. Despite the lower accumulation of hepatic glycogen in the fed state, glycogen depletion was not impaired during fasting in *Iqgap2*^*−/−*^ mice. These data imply a defect in synthesis rather than the breakdown of glycogen. Immunoprecipitation experiments show IQGAP2 can scaffold GYS2 and GSK3. Similarly, another scaffold, protein targeting to glycogen, was shown to regulate phosphorylase kinase, phosphorylase a, and GYS2 to control glycogen levels (36). In addition, protein targeting to glycogen, PTG heterozygous mice showed decreased GYS2 activity (37). These results support that scaffold proteins, including IQGAP2, may facilitate hepatic glycogenesis.

IQGAP2 SNP variants are strongly associated with diabetic phenotype, and IQGAP2-deficient mice have been reported to exhibit conflicting insulin effects (19, 20, 21). To thoroughly examine the role of IQGAP2 on the insulin signaling pathway, we depleted IQGAP2 in a liver cell line. We find that the knockdown of IQGAP2 was sufficient to increase IRS1 protein levels and decrease AKT phosphorylation upon insulin stimulation. In a recent study, IQGAP1 was found to bind IRS1 directly *via* its C terminal (containing the RGCT domain) and decrease insulin-stimulated phosphorylation of AKT (32). Since the RGCT domain of IQGAP2 shares 89% similarity with IQGAP1 (15), we speculate that IQGAP2 may similarly influence AKT phosphorylation.

FOXO proteins are direct targets of insulin signaling and are negatively regulated downstream of the PI3K/AKT pathway. FOXO1 and FOXO3 synergistically regulate glucose and lipid metabolism in the liver. AKT phosphorylation of FOXO3a at the Thr32 site leads to its degradation (38). Consistently, we find that IQGAP2-depleted HepG2 exhibits lower AKT activation with a concomitant decrease in FOXO3a phosphorylation (at the Thr32 site). Furthermore, FOXO1 and FOXO3 overexpression inhibits the transcription of *Srebp1c* and fatty acid synthesis gene (39, 40). This is interesting as we do find reduced expression of *Srebp1c* and *Fasn* in the *Iqgap2*^*−/−*^ livers. Taken together, these findings implicate that IQGAP2 is necessary for maintaining insulin signaling, AKT activation, and FOXO inactivation in the postprandial state.

Some limitations of this study are that we did not investigate domain-specific interactions of IQGAP2. For instance, is a specific domain of IQGAP2 necessary to complex with several members simultaneously downstream of insulin receptor (IRS1, PIP3, and AKT) and to glycogen synthesis (GSK3 and GYS2), or does it interact with these proteins using distinct domains remains to be elucidated. Additionally, we found a prominent disruption in energy homeostasis in *Iqgap2*^*−/−*^ female livers, although IQGAP2 expression was not responsive to estrogen signaling. Future studies will be required to address the sex-specific role of IQGAP2 in maintaining fed state metabolism.

In summary, we find that IQGAP2 can interact with effectors of the insulin signaling pathway, and the absence of this scaffold reduced the expression of genes involved in lipogenic and glycogen synthesis in mouse livers. Overall, these findings suggest IQGAP2 facilitates the insulin downstream cascade, and its absence results in defective fed state metabolism in the liver.

## Experimental procedures

### Animal experiments

The generation of *Iqgap2*^*−/−*^ mice has been previously described (41). Wildtype (129/SVJ) and *Iqgap2*^*−/−*^ mice were obtained from Valentina Schmidt, Stony Brook University. Wildtype control mice were derived from heterozygous *Iqgap2*^*+/−*^ mice as *Iqgap2*^*+/+*^mice. The original 129/SVJ was derived from the heterozygous *Iqgap*2+/−. We have used homozygous −/− and +/+ litter to increase the number of−/− and +/+ originally derived from the het/het mice. The mice were maintained on a 129/SVJ background and were housed in flow cages at 24 °C on a 12/12-hour-light/dark cycle, with lights on starting at 6 AM CST, corresponding to zeitgeber time (ZT) 0. Female and male 16 to 20-week-old mice were used for all experiments. WT and *Iqgap2*^*−/−*^ mice were randomly divided into ad libitum-fed group or a 24-h fasting group. Fed mice were placed on a standard chow diet (Teklad F6 Rodent Diet, 8664) and allowed ad libitum access to food and water. For the 24-h fasting experiments, mice were placed in new cages without food, initiating fasting at 9 AM (ZT4) before the day of sacrifice. All mice were sacrificed at ZT4-6. This study was not blinded. Genotype was confirmed by PCR analysis of genomic DNA (n = 4–10 mice per genotype) as previously described (41). A portion of each tissue was fixed in 10% formalin for histological analysis.

### Cell culture and transfection

HepG2 cells and HEK293T were obtained from the American Type Culture Collection (ATCC) and cultured following ATCC specifications. For IQGAP2 knockdown, HepG2 cells were seeded at 30% confluency and transduced with shRNA lentivirus produced from plasmid DNA for human IQGAP2 (Mission TRCN0000414490, TRCN0000047493, TRCN0000430206; Sigma-Aldrich) and 8ug/ml polybrene. After 24 h, transduced cells were selected using 1ug/ml puromycin. After 72 h of puromycin selection, IQGAP2 knockdown efficiency was assessed by isolating protein from cells and running Western blots. IQGAP2 protein expression levels were evaluated by immunoblotting with mouse anti-IQGAP2.

### Insulin stimulation

HepG2 cells were serum starved overnight, followed by 100 nM insulin stimulation for 5, 10, and 30 min (42). Protein lysate was collected immediately for analysis. Each experiment was repeated two to eight times in triplicates.

### E2 and EE2 stimulation

HepG2 cells with stable transfection of ERα (43) were cultured in charcoal-stripped 10% FBS-DMEM for 5 days, followed by 1µM E2 and 0.5 and 1 µM EE2 stimulation. After 24 hours of treatment, cells were washed with 1X PBS, and RNA was collected using TRIzol solution (Ambion).

### Glycogen measurement

Hepatic glycogen was measured using Abcam Glycogen Assay Kit II (colorimetric). In brief, 10 mg liver samples were homogenized in 200 μl H2O. First, the collected supernatant was boiled to inactivate enzymes. Then, hydrolysis enzyme and buffer were added to digest glycogen to glucose, and the probe was added for colorimetric quantification of glucose concentration. Finally, the sample background (glucose present before glycogen hydrolysis) was subtracted for each sample.

### Hepatic triglyceride measurement

Liver tissue (10–25 mg) was homogenized in chilled hexanes/isopropanol (3:2) solvent. The liquid phase was collected, and the sample was allowed to dry overnight. Triglycerides were resolubilized in PBS-2%Triton X-100 mixture and measured using Infinity Triglyceride Liquid Stable Reagent (Fischer Scientific).

### Histology

Formalin-fixed liver sections were stained to assess general histology (hematoxylin and eosin), immunohistochemistry, and glycogen content [PAS and PAS/D (PAS with Diastase)] intensity levels. Methods for each stain are described in detail below.

### Hematoxylin and eosin stain

Formalin-fixed liver samples were embedded in paraffin wax, and five-micron sections were cut. The tissue samples were deparaffinized with xylene (3 times for 5 min) and hydrated through a series of decreasing concentrations of ethanol (3 washes of 100% ethanol, followed by 95%, 80%, and 50% ethanol for 3 min each). The samples were then placed in distilled water for 1 min before being stained using Hematoxylin 7211 (Richard-Allan Scientific) for 2 min and bluing reagent (0.1% sodium bicarbonate) for 1 min. Slides were placed under running DI water in between both steps until clear. Slides were then placed in 95% ethanol for 2 min before being placed into Eosin-Y (Richard-Allan Scientific) for 25s. Finally, the samples were dehydrated in three washes of 100% ethanol before being cleared in xylene. Samples were left in xylene overnight. Coverslips were mounted using Permount mounting media (Fisher Chemical). Images were taken using EVOS Microscope (AMG) at 20× magnification, using Bright-Field microscopy.

### PAS stain

Formalin-fixed liver samples were deparaffinized and hydrated to water, as listed above. Each PAS-labeled sample slide had a corresponding PAS/D-labeled sample slide. Diastase is an enzyme that breaks down glycogen, and PAS/D samples were used as controls. The PAS/D-labeled samples were immersed in distilled water for 2 min before being placed into a 50 °C water bath with the diastase (with a-amylase) solution. The diastase solution was made by combining 0.05 g diastase (MP Biomedicals) with 50 ml of phosphate buffer (0.35 g sodium phosphate, dibasic (Na2HPO4); 3.5 g sodium phosphate, monobasic (NaH2PO4); 200 ml water; and pH adjusted to 5.8). PAS/D-labeled slides were left in diastase solution for 1 h, while their corresponding PAS-labeled slides were left in distilled water for the same time. The PAS/D-labeled slides were then rinsed in running tap water and immersed in distilled water for 2 min. Next, all slides were oxidized in 0.5% periodic acid solution (0.5 g periodic acid (Sigma-Aldrich) in 100 ml of distilled water) for 5 min and rinsed in distilled water. Slides were placed in Schiff Reagent (Acros Organics; Catalog: 611175000) for 15 min in a foil-covered container and washed in lukewarm tap water for 5 min. Sections transitioned from light pink to dark pink at this stage, indicating the Schiff Reagent. Samples were counterstained using Hematoxylin 7211 for 20 s and washed in tap water for 5 min. Samples were dehydrated in three washes of 100% ethanol before being cleared in xylene. Samples were left in xylene overnight. Coverslips were mounted using Permount mounting media. Images were taken using EVOS Microscope (AMG) at 20× magnification, using Bright-Field microscopy.

### Immunohistochemical analysis

As listed above, formalin-fixed tissue samples were deparaffinized and hydrated in distilled water. Antigen retrieval was performed using a Tris-EDTA buffer and microwaving slides for 35 min. After cooling, slides were rinsed in tap water and washed twice with 1X TBS (0.025% Triton). Slides were washed, blocked, and then incubated with primary antibody IQGAP2, GS, or E-cadherin (1:100 dilution) overnight at 4 °C; slides were then incubated with secondary antibody (1:250 dilution) for 1 h at room temperature. Finally, slides were washed and counterstained with TO-PRO-3 Iodide (642/661) (Thermo Fisher Scientific). Coverslips were mounted using CC Mount. Images were taken using Zeiss LSM 700 Confocal microscope at the Carl R. Woese Institute for Genomic Biology at UIUC.

### Western blot analysis

Protein was isolated and homogenized from frozen whole liver tissue through sonification in RIPA buffer (25 mm Tris pH 7–8; 150 mm NaCl; 0.5% Na deoxycholate; 1% Triton X-100; 0.1% SDS; 0.1% phosphatase inhibitor; one tablet protease inhibitor; in water). The supernatant was collected, and protein concentration was measured using a BCA protein assay kit (Thermo Scientific). For Western blots, 30 to 50 μg of sample protein was run through a 5% stacking gel (40% acrylamide; 1.5 M Tris pH 6.8; 10% SDS; 10% APS; water; and TEMED) and then 8% resolving gel (40% acrylamide; 1.5 M Tris pH 8.8; 10% SDS; 10% APS; water; and TEMED) at 70 V for 3 h. Next, proteins were transferred from the gel to a PVDF membrane at 35V overnight at 4 °C. Membranes were stained with Ponceau S, rinsed with water, and imaged to detect protein bands. Afterward, membranes were washed with 1× TBST and blocked with blocking buffer (5% milk in TBST) for 1 h on the shaker. Membranes were then incubated in primary antibodies (5% BSA buffer or 5% milk in TBST) overnight at 4 °C on the shaker. Next, secondary antibodies (5% milk in TBST) were added, and membranes were incubated for 1 h on the shaker at room temperature. Membranes were washed with TBST between steps. Target proteins were detected using SuperSignal West Femto Luminol/Enhancer solution and SuperSignal West Femto Peroxide Buffer (Thermo Scientific). Information about primary and secondary antibodies used is listed in Table S1. Complete unedited blots are shown in Fig. S7.

### Immunoprecipitation

Hek293T cells or liver tissue samples were lysed in 1 ml 1% NP-40 lysis buffer containing protease inhibitor (25 Mm Hepes, pH 7.8, 150 mM NaCl, 5 mM EDTA, pH 8.0, 0.5 mM CaCl2, 1.0% NP-40). The lysate was then split, and equal amounts were immunoprecipitated with protein A/G magnetic beads (Thermo Fisher Scientific) [prebound with IQGAP2 (ab181127, 3 μg) or IgG (Invitrogen 02-610-2, 3 μg) antibody] overnight, washed, and resolved on an 8% SDS-PAGE gel. Blots were probed with antibodies for IQGAP2, GYS2, GSK3, and AKT.

### Quantitative real-time PCR analysis

RNA from frozen whole liver tissue was isolated using TRIzol solution (Ambion) based on the manufacturer’s protocol. RNA quality was determined using A260/280 and bleach RNA gel as previously described (44). RNA (3 μg) was treated with DNase (New England Biolabs) and reverse transcribed using random primer mix (New England Biolabs) and the Maxima Reverse Transcriptase kit (Thermo Fisher Scientific). The cDNA was diluted to 12.5 ng/μl with molecular-grade water (Corning) and used for qRT-PCR assays. qRT-PCR was performed on an Eco Real-Time PCR system (Illumina) in triplicates using PerfeCTa SYBR Green FastMix (Quanta). All assays were run with an initial activation step for 10 min at 95 °C, followed by 40 cycles of 95 °C for 15 s and 60 °C for 1 min. *Tbp* was used as the housekeeping gene. Primer sequences of genes used are listed in Table S2.

### Statistical analysis

All statistical analyses were performed using GraphPad Prism software. Student’s unpaired 2-tailed *t* test was used to compare two groups. Welch’s *t* test was performed to compare two groups with unequal variance. A two-way ANOVA with Bonferroni multiple comparisons test was performed to compare two groups with two treatments. Mean ± SD was plotted. Significance was determined by *p* <0.05. Outliers were determined using Grubbs’ test and removed from the analysis.

### Study approval

All animal studies were approved by the University of Illinois at Urbana-Champaign Institutional Animal Care and Use Committee.

## Data availability

All data provided are contained within this manuscript.

## Supporting information

This article contains supporting information.

## Conflict of interest

The authors declare that they have no conflicts of interest with the contents of this article.
