# Supplementary material for: Depletion of IQ motif-containing GTPase activating protein 2 (IQGAP2) reduces hepatic glycogen and impairs insulin signaling
Source: J Biol Chem. 2023 Oct 5;299(11):105322. doi: 10.1016/j.jbc.2023.105322 (PMC10652104; doi:10.1016/j.jbc.2023.105322)
Supplement: Supporting Figures S1–S8 and Tables S1 and S2 [file mmc1.docx]

**Supporting information**


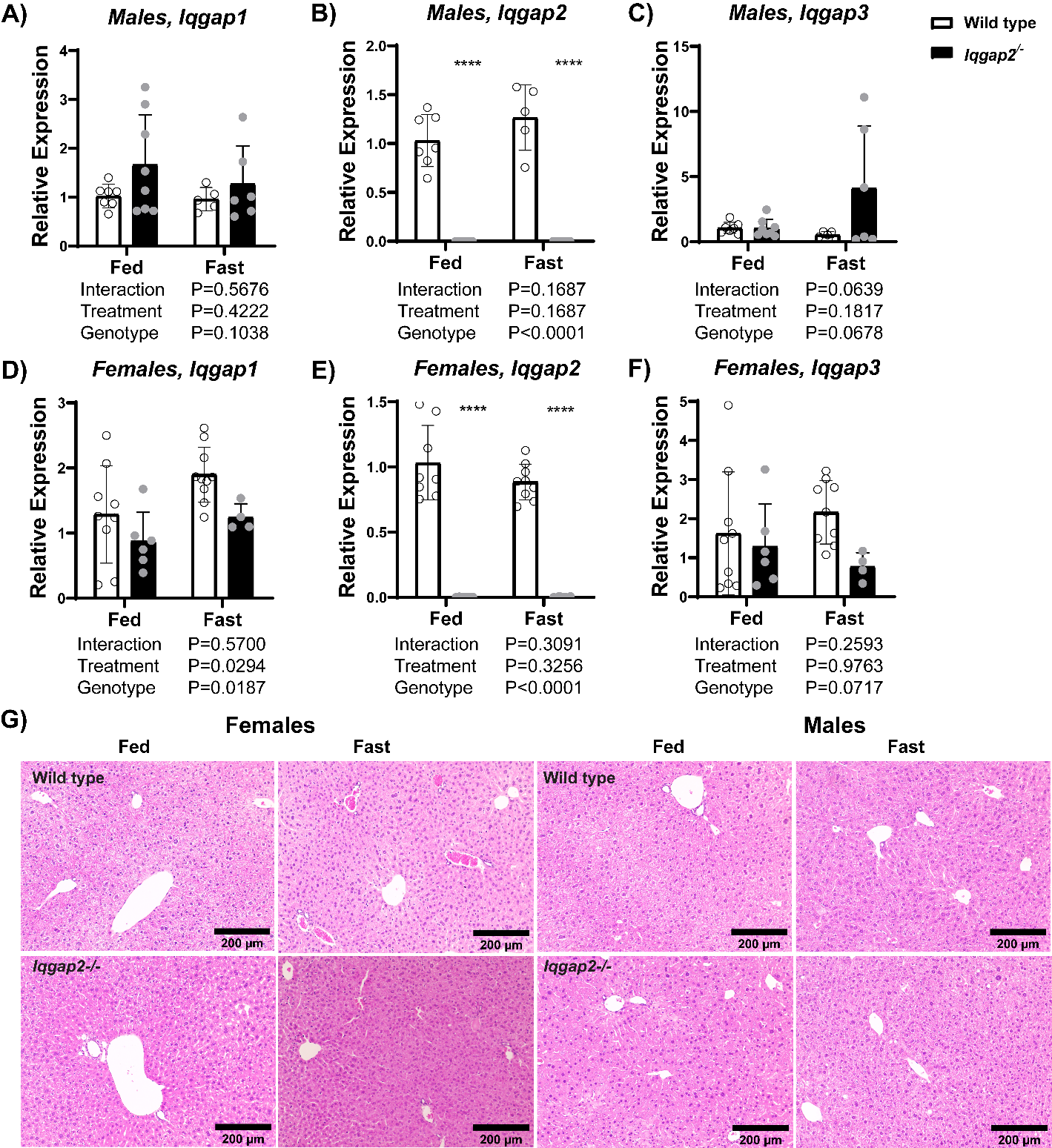


***Fig. S1. Expression of various Iqgap genes and overall liver architecture is unaltered in Iqgap2^-/-^ mice.*** *Iqgap2^-/-^* and WT mice (males and females, 16-20 weeks old, n=5-9) were fed regular chow ad libitum or fasted for 24 hours. Hepatic mRNA transcript levels of *Iqgap1,* *Iqgap2,* and *Iqgap3* were determined using qRT-PCR. The absence of IQGAP2 did not alter *Iqgap1* (A, D) and *Iqgap3* transcript expression (C, F), and fasting did not induce *Iqgap2* transcript expression (B, E). Liver tissues were sectioned and stained for histological examination using hematoxylin and eosin. No overt inflammation or changes in hepatocyte size were observed in either (G) female or male livers. N=3-5 per group. Bar, 200μm. Statistics were calculated using two-way ANOVA with Bonferroni post hoc analysis. ****P<0.0001 vs. WT control.


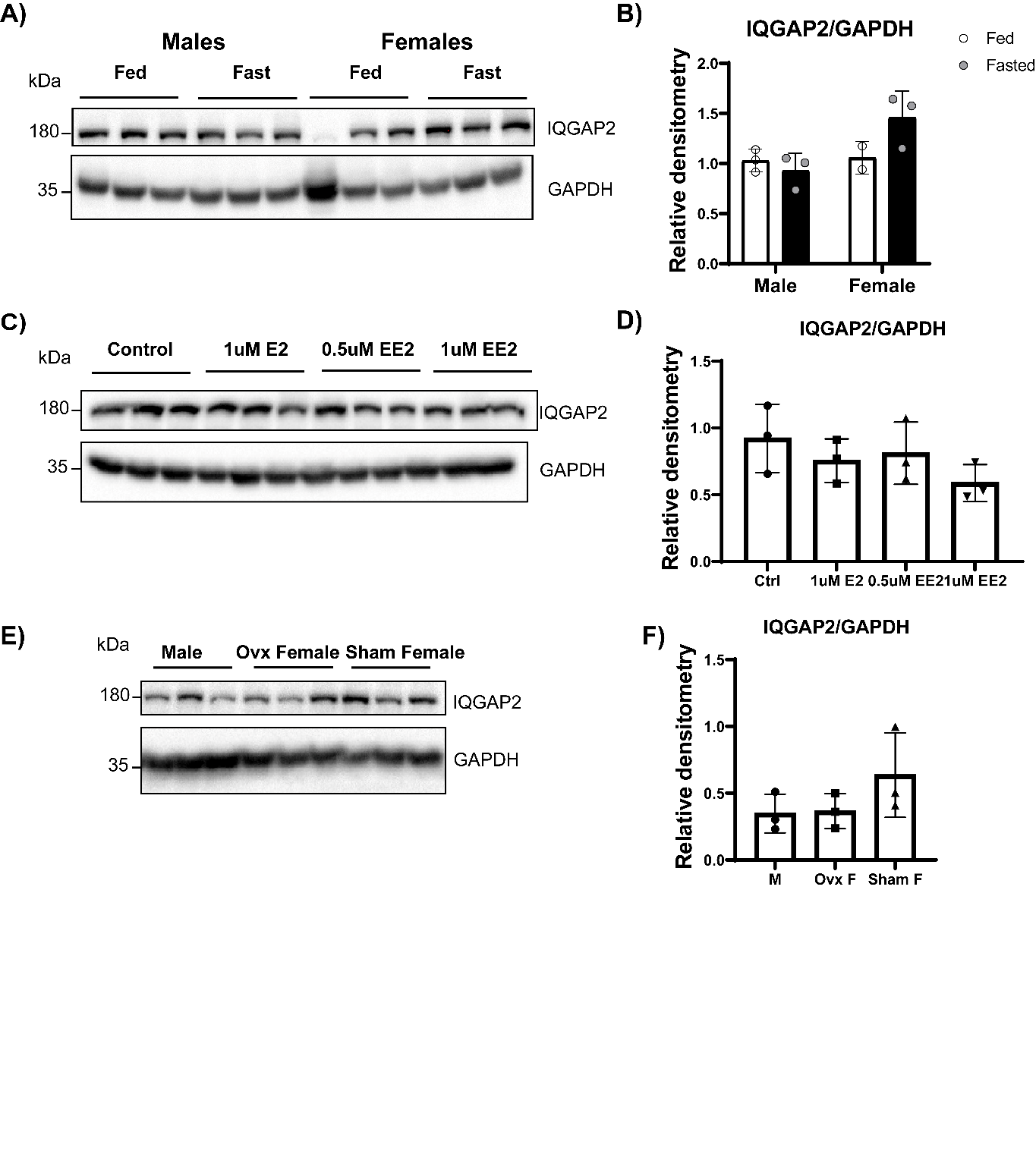


***Fig. S2.* IQGAP *expression is not sex-specific.*** WT mice (males and females, 16-20 weeks old) were fed regular chow ad libitum or fasted for 24 hours. Liver tissue was harvested, and hepatic expression of IQGAP2 protein levels was determined using western blots. Similar expression of IQGAP2 protein was noted in both sexes (A, B). To examine whether estrogen signaling could regulate IQGAP2 expression, we treated ERα expressing HepG2 cells with E2 or EE2 (C) and found IQGAP2 was not induced (D). In addition, we examined control and ovariectomized (Ovx) livers (E). IQGAP2 expression was not significantly altered (F). Statistics were calculated using one-way or two-way ANOVA with Bonferroni post hoc analysis. E2, estradiol; EE2, ethinyl estradiol.


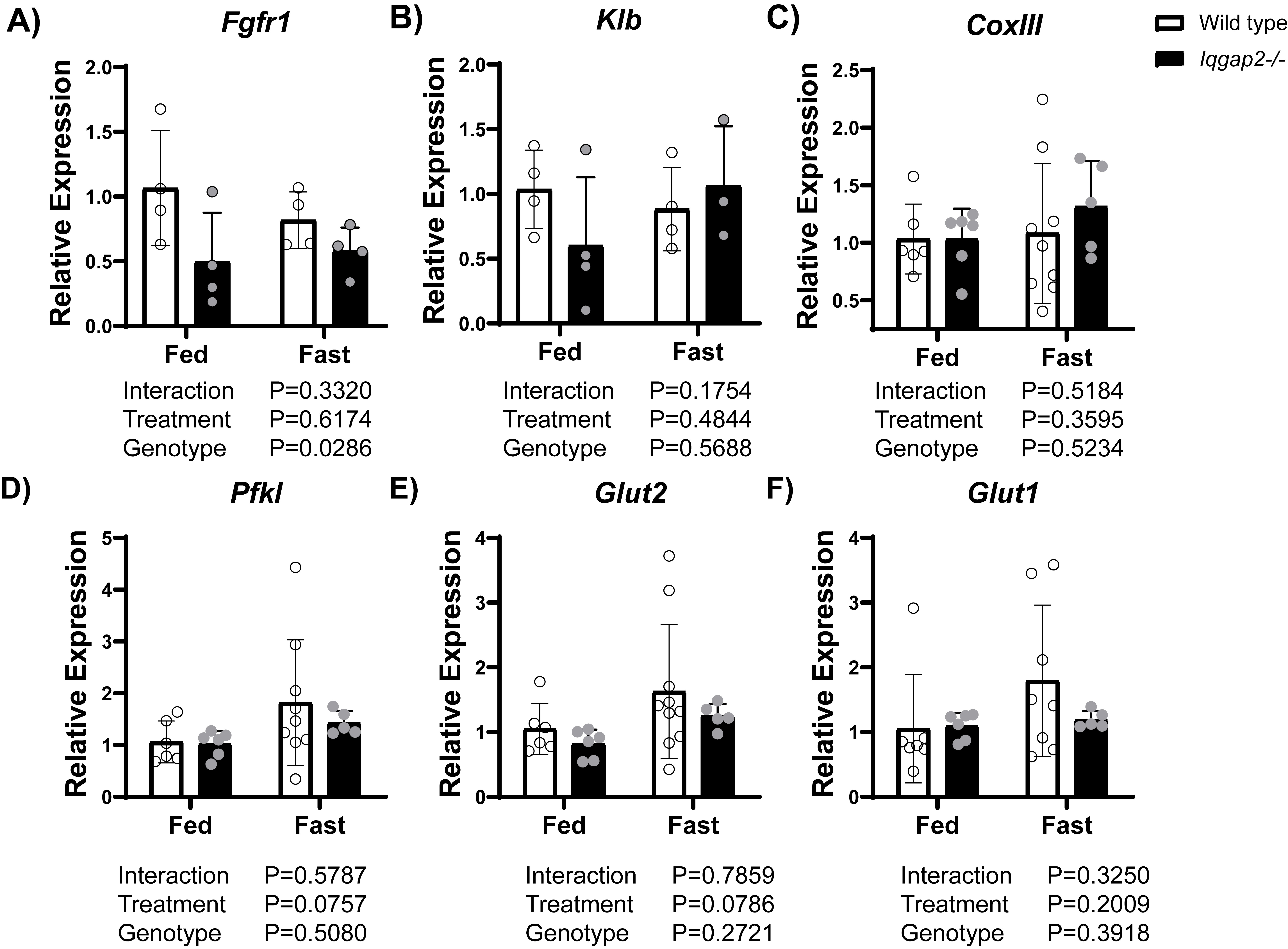


***Fig. S3. Iqgap2^-/-^ livers do not exhibit defects in fasting response receptors, mitochondrial, glycolysis, and glucose uptake genes.*** Enterokine receptor (A) *Fgfr1* and coreceptor (B) *Klb* gene expression was quantified using qRT-PCR. Fed state levels were comparable in *Iqgap2^-/-^* female mice. Mitochondrial complex III (C) transcript levels were similar between the groups. *Iqgap2^-/-^* mice showed no difference in the expression of glycolysis regulator (D) phosphofructokinase and glucose uptake transporters (E, F). Statistics were calculated using two-way ANOVA with Bonferroni post hoc analysis. N= 5-9 mice per group.


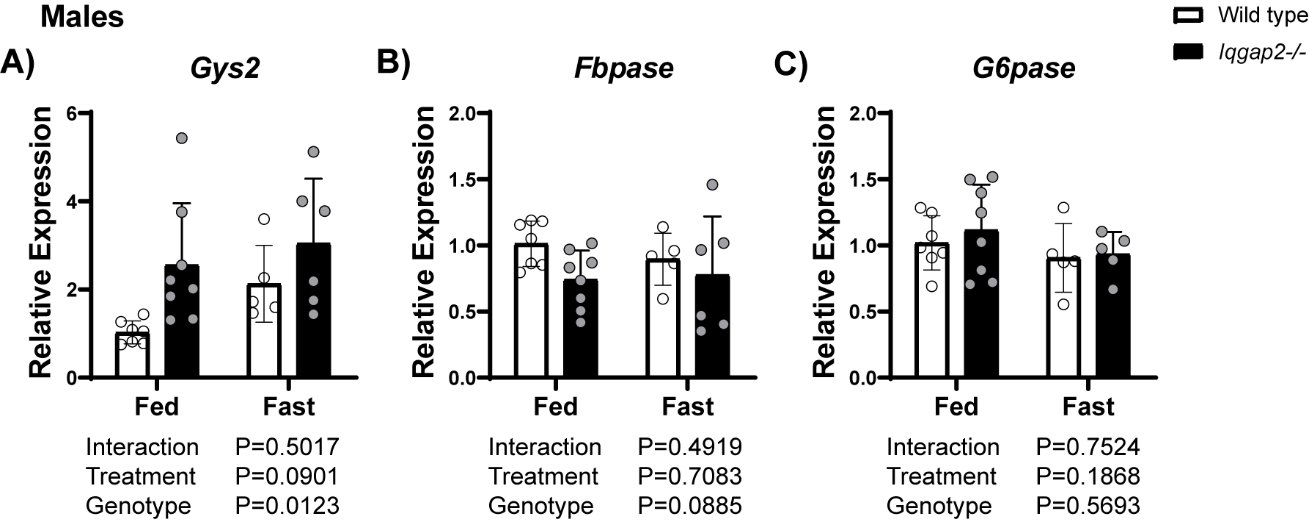


***Fig. S4. Carbohydrate metabolism is unaltered in male Iqgap2^-/-^ mice.*** Liver tissues from male *Iqgap2^-/-^* and wild-type mice were examined to quantify the expression of genes involved in glycogen synthesis and gluconeogenesis. Glycogen synthase gene expression showed an increasing trend in *Iqgap2^-/-^* male mice (A). However, gluconeogenic gene expression was unaltered in both genotypes (B, C). Statistics were calculated using two-way ANOVA with Bonferroni post hoc analysis.


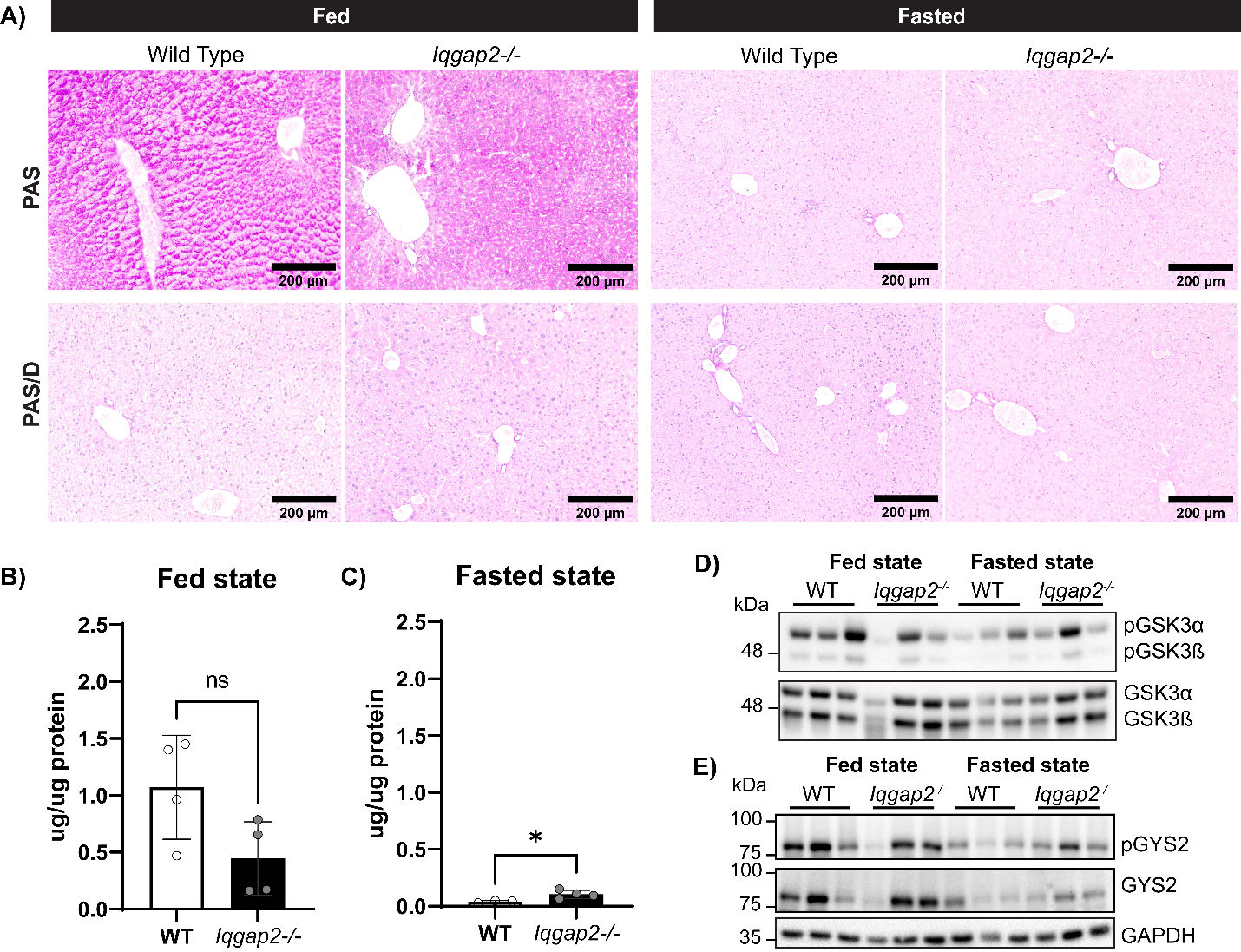


***Fig. S5. Male Iqgap2^-/-^ mice exhibit a modest decrease in hepatic glycogen storage.*** Male *Iqgap2^-/-^* and WT mice were fed regular chow ad libitum. Liver tissue was histologically stained with Periodic Acid-Schiff’s base (PAS) to examine glycogen levels. Hepatic glycogen in (A) fed mice was uniformly distributed with a modest decrease of glycogen storage around the periportal region in *Iqgap2^-/-^* mice and was completely mobilized in the 24-hour fasted state. Biochemical quantification showed a similar reducing trend in the hepatic glycogen only in the fed *Iqgap2^-/-^* mice (B, C). Hepatic total GSK3α/β and phosphorylated GSK3α/β (ser21/9) (D) and total GYS2 and phosphorylated GYS2 (E) protein levels were measured by western blots. Protein expression was comparable between male *Iqgap2^-/-^* and WT mice. N=3-5 mice per group. Bar, 200μm.


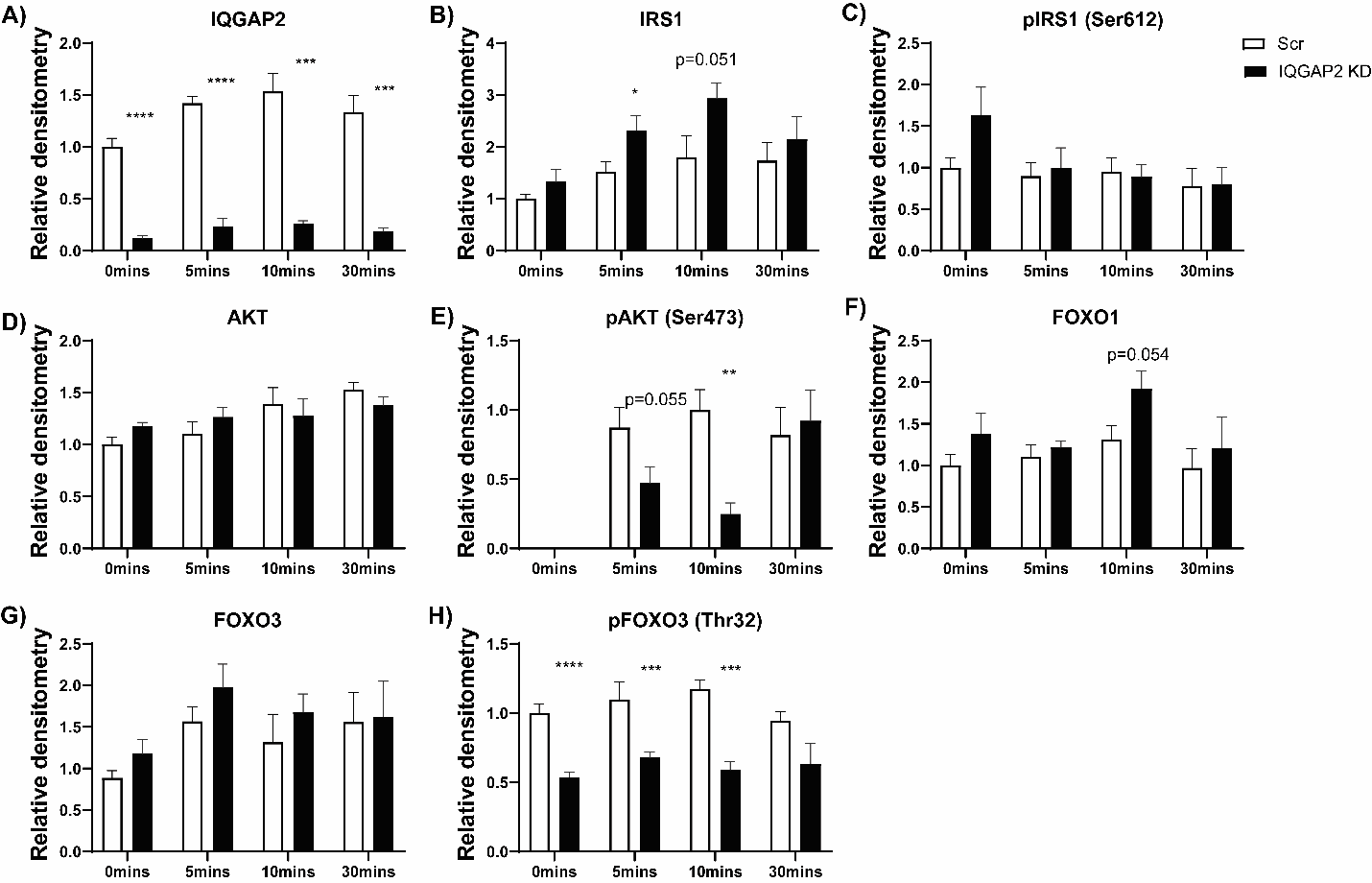


***Fig. S6.* IQGAP2 *knockdown impairs insulin downstream signaling.*** HepG2 liver cells were transfected with scrambled or IQGAP2 shRNA before being treated with insulin (100nM for 5,10, or 30 minutes) **t**o examine whether IQGAP2 blunts the insulin response. Western blot densitometric analysis (A-H) examined changes in the insulin signaling pathway. Scr- Scramble shRNA, KD- knockdown with IQGAP2 shRNA. Glyceraldehyde-3- phosphate dehydrogenase (GAPDH) served as a loading control for immunoblot analysis. Values are displayed as mean±SEM. Statistics were calculated using Student’s T-test analysis. *P<0.05, **P<0.01, ***P<0.001, ***P<0.0001.


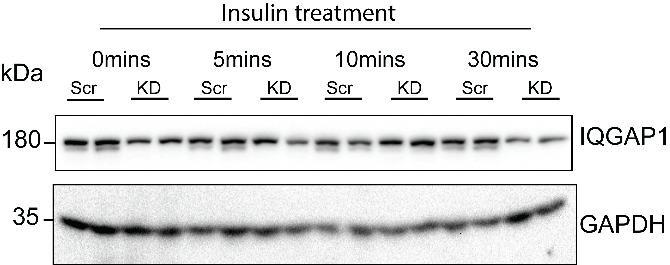


***Fig. S7.* IQGAP1 *levels are maintained in* IQGAP2 *knocked-down cells***. HepG2 liver cells were transfected with scrambled or IQGAP2 shRNA before being treated with insulin (100nM for 5,10, or 30 minutes). IQGAP1 protein levels were measured to investigate whether altered IQGAP1 expression contributed to the impaired insulin pathway observed in IQGAP2-depleted cells. IQGAP1 protein levels were unchanged. Scr- Scramble shRNA, KD- knockdown with IQGAP2 shRNA. Glyceraldehyde-3- phosphate dehydrogenase (GAPDH) served as a loading control for immunoblot analysis.

***Fig. S8. Full unedited gels.***

For Fig. 5A.

pGSK3α/β GSK3α/β


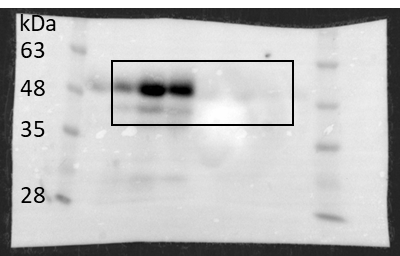

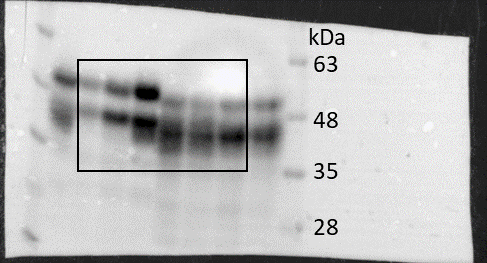


For Fig. 5B.

pGYS2 GYS2 GAPDH


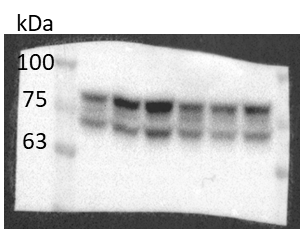

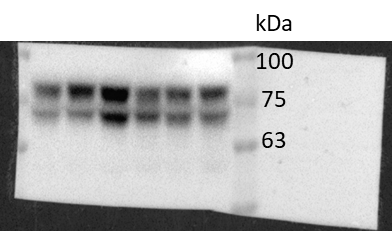

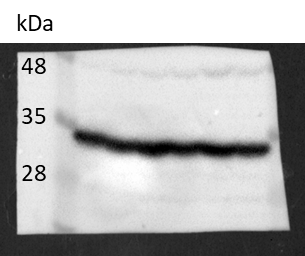


For Fig. 5C.

IQGAP2 GSK3α/β GYS2


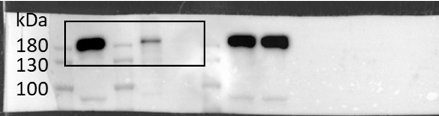

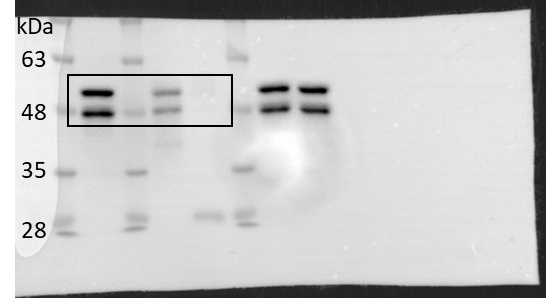

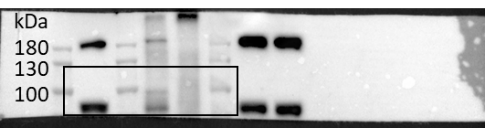


For Fig. 5D.

IQGAP2 GYS2


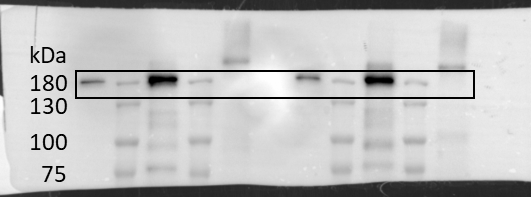

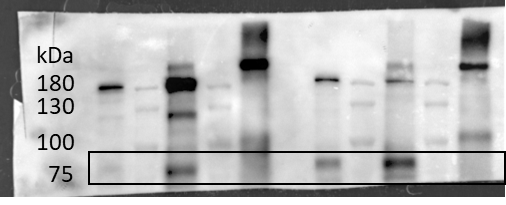


For Fig6A

IQGAP2 GAPDH


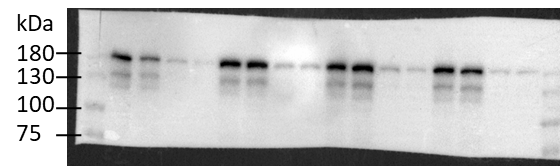

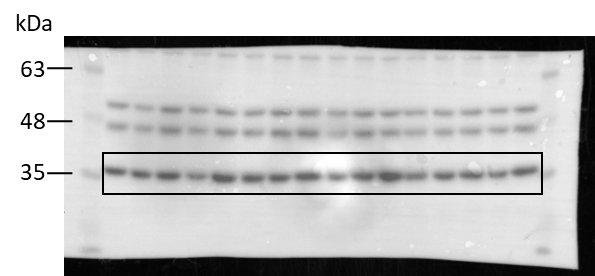


IRS1 pIRS1(S612)


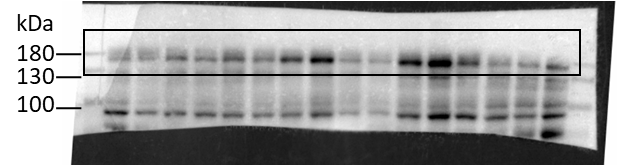

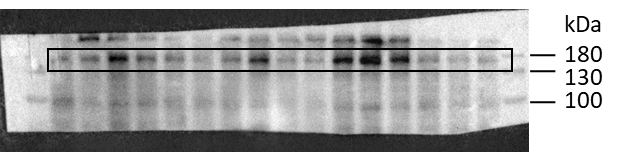


AKT pAKT (S473)


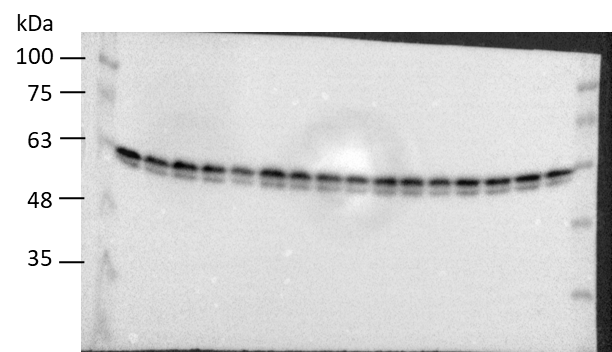

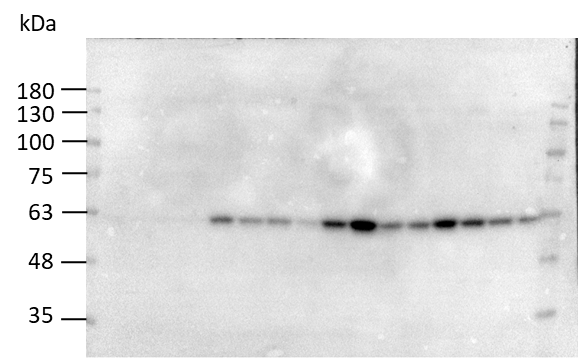


FOXO3 pFOXO3(Thr32)


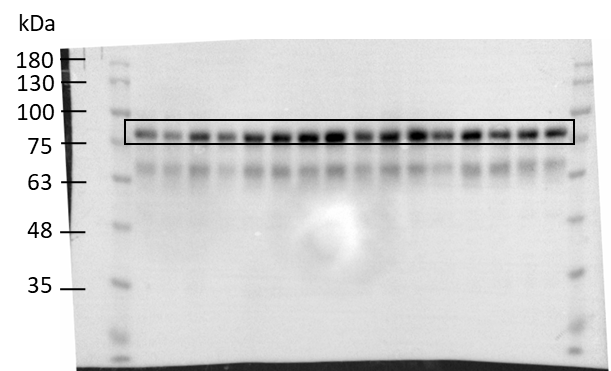

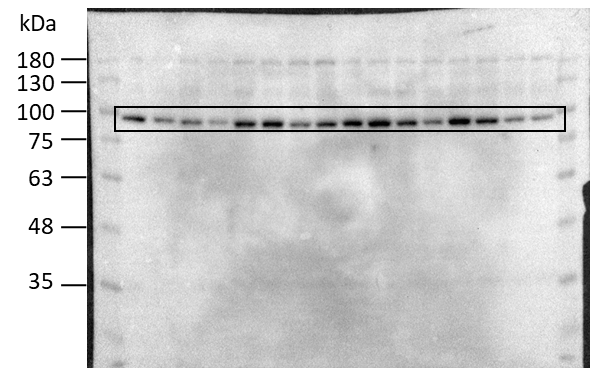


FOXO1


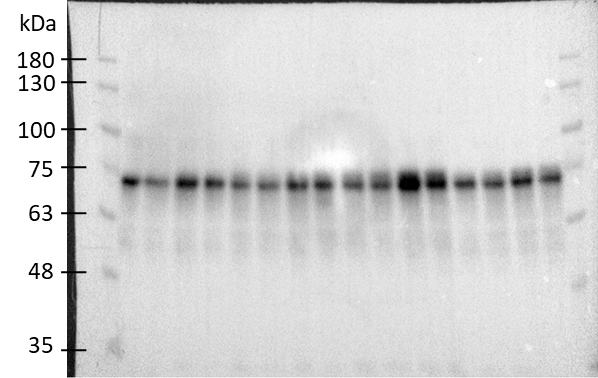


Full Unedited Gels for Supplementary Figures

For Fig. S2A.

IQGAP2


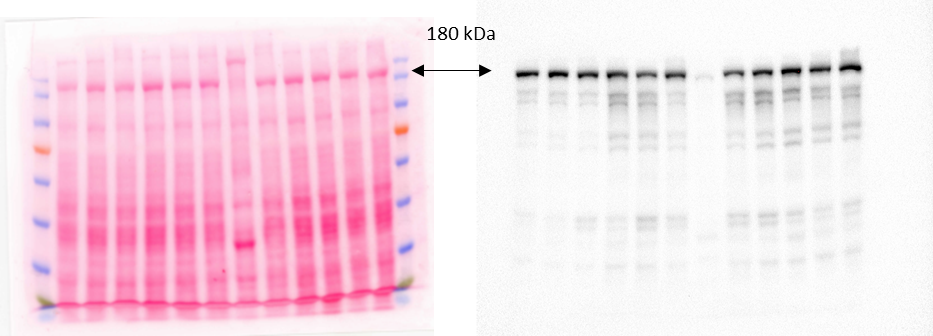


GAPDH


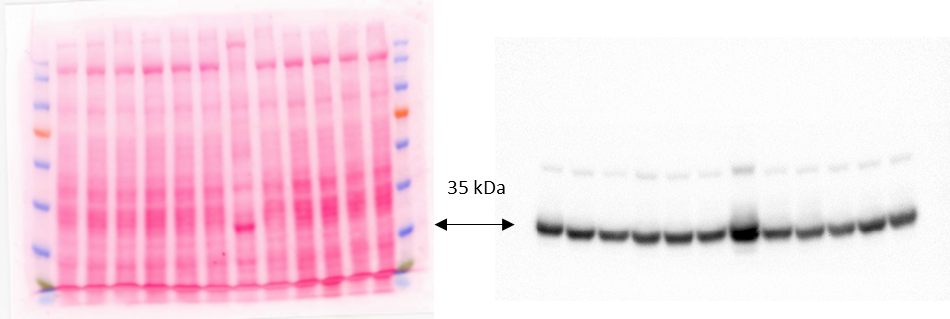


For FigS2C.

IQGAP2 GAPDH


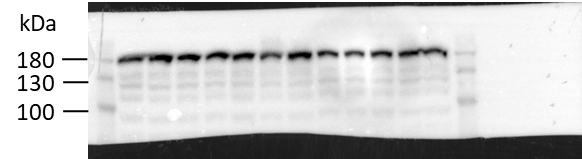

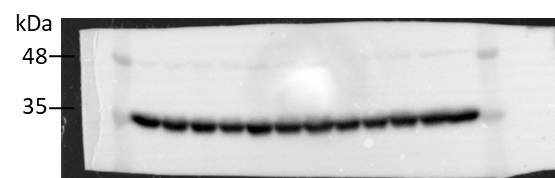


For FigS2E.

IQGAP2 GAPDH


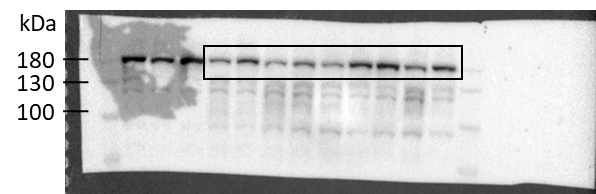

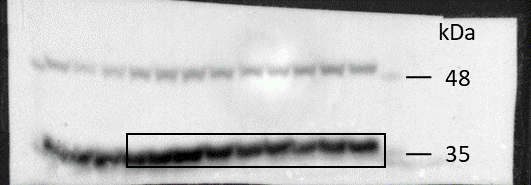


For FigS5D.

GSK3α/β pGSK3α/β


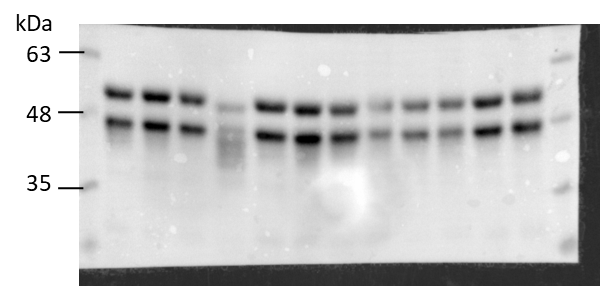

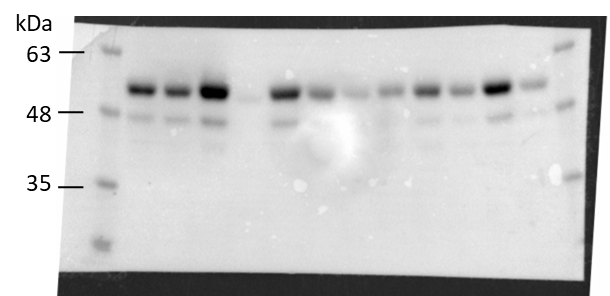


For FigS5E.

GYS2 pGSY2


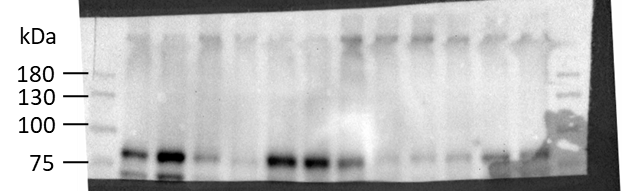

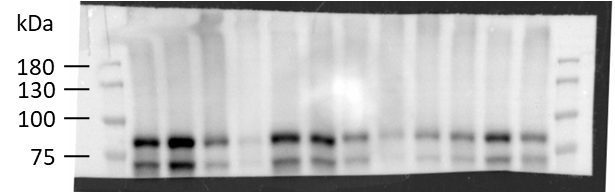


GAPDH


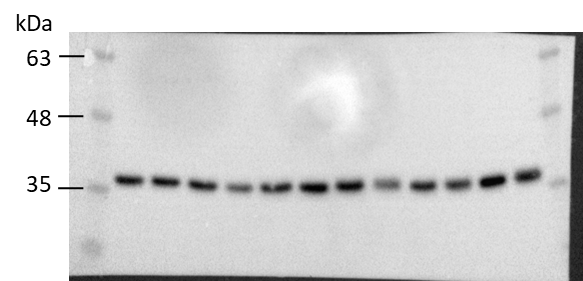


For FigS7.

IQGAP1 GAPDH


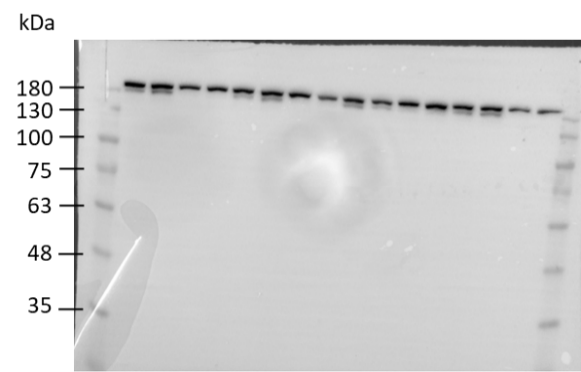

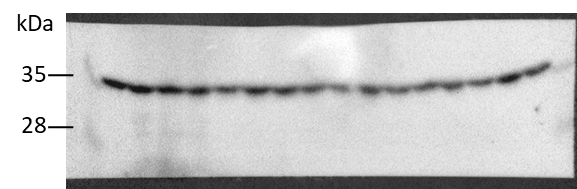


**Table S1. Antibodies used for western blots and immunohistochemistry.**

| Antibody | Source | Catalog number | RRID |
| --- | --- | --- | --- |
| *Primary antibodies* |  |  |  |
| IQGAP2 Rabbit mAb | Abcam | ab181127 | AB_2833247 |
| IQGAP2 Mouse mAb | Santa Cruz Biotechnology | Sc-17835 | AB_2125909 |
| IQGAP1 antibody [EPR5220] | Abcam | ab133490 | AB_11157518 |
| GSK-3α/β (D75D3) XP Rabbit mAb | Cell Signaling Technology | #5676 | AB_10547140 |
| GSK-3α/β Mouse mAb | Santa Cruz Biotechnology | Sc-7291 | AB_2279451 |
| Phospho-GSK-3a/B (Ser21/9) (D17D2) Rabbit mAb | Cell Signaling Technology | #8566 | AB_10860069 |
| Glycogen Synthase Rabbit mAb | Cell Signaling Technology | #3886 | [AB_2116392](http://antibodyregistry.org/AB_2116392) |
| Phospho-Glycogen Synthase (Ser641) | Cell Signaling Technology | #3891 | [AB_2116390](http://antibodyregistry.org/AB_2116390) |
| GAPDH Rabbit | Sigma | G9545 | AB_796208 |
| IRS-1 (D23G12) Rabbit mAb | Cell Signaling Technology | #3407 | AB_2127860 |
| Phospho-IRS-1 (Ser612) (C15H5) Rabbit mAb | Cell Signaling Technology | #3203 | AB_1031167 |
| Akt (pan) (C67E7) Rabbit mAb | Cell Signaling Technology | 4691S | AB_915783 |
| Phospho-Akt (S473) (D9E) XP(R) Rabbit mAb | Cell Signaling Technology | 4060S | AB_2315049 |
| FoxO1 (C29H4) Rabbit mAb | Cell Signaling Technology | #2880 | AB_2106495 |
| FoxO3a (D19A7) Rabbit mAb | Cell Signaling Technology | #12829 | AB_2636990 |
| Phospho-FoxO1 (Thr24)/FoxO3a (Thr32)/FoxO4 (Thr28) (4G6) Rabbit mAb | Cell Signaling Technology | #2599 | AB_2106814 |
| Rabbit IgG Isotype Control | Invitrogen | 02-610-2 | AB_2532938 |
| *Secondary antibodies* |  |  |  |
| Goat anti-Mouse | ThermoFisher Scientific | #35503 | 1965946 |
| Goat anti-Rabbit | ThermoFisher Scientific | #31460 | AB_228341 |

**Table S2. Primer sequences used for real-time qRT-PCR.**

| Gene | Forward primer (5’- 3’) | Reverse primer (5’- 3’) |
| --- | --- | --- |
| Scaffold protein |  |  |
| *Iqgap1* | CATCAACACCCACACTCTCC | GTCGTTTGAATTGCCACAGC |
| *Iqgap2* | TCAAGATTGGACTGCTGGTG | AGGTTTGGTCTGGAGGAGGT |
| *Iqgap3* | CTCTGGTCACCTTGCAGAAT | CAGCAGCTCTTGGTAGACAG |
| Lipid metabolism |  |  |
| *Cpt1a* | TGATGACGGCTATGGTGTTTC | CAAACAAGGTGATAATGTCCATC |
| *Dgat2* | CTCTGTCACCTGGCTCAACA | TATCAGCCAGCAGTCTGTGC |
| *Fasn* | GCTGCGGAAACTTCAGGAAAT | AGAGACGTGTCACTCCTGGACTT |
| *Srebp1c* | GGAGCCATGGATTGCACATT | GGCCCGGGAAGTCACTGT |
| Carbohydrate metabolism | |  |
| *Fbpase* | TGTGGGCTCCACCTGCCTGCACCTTTAGTC | TTTGATCGCGGTGCAGAGCGAATTCAGCAG |
| *G6pase* | TTTATGGGTTGACTGCTCTGG | AAAGATCTAGGCCCAGTGTAAAG |
| *Glut1* | TGGGCAAGTCCTTTGAGATG | TGACACCTCTCCCACATACA |
| *Glut2* | CTGTTCCTAACCGGGATGAT | ATCCAGGCGAATTTATCCAG |
| *Gys2* | CCATCCTCAGCACCATTAGAC | GTGACAACCTCGAACAAACTC |
| *Pfkl* | CACTGGGACAACATGACCAG | GCAATGGGATCTGATGACCT |
| Fasting signal |  |  |
| *Fgfr1* | CAACCGTACCCGCATCAC | TACGGAGAAGTAGGTGGTATC |
| *Fgf21* | TGGATCGCCTCACTTTGATCC | CCATGGGCTTCAGACTGGTA |
| *Klb* | GATGAAGAATTTCCTAAACCAGGTT | AACCAAACACGCGGATTTC |
| Mitochondrial function | |  |
| *CoxII* | TGAAGACGTCCTCCACTCATGA | GCCTGGGATGGCATCAGTT |
| *CoxIII* | GCAGGATTCTTCTGAGCGTTCT | GTCAGCAGCCTCCTAGATCATGT |
| *Pgc1a* | CCCACAGAAAACAGGAACAG | CTGGGGTCAGAGGAAGAGAT |
